# The *afc* antifungal activity cluster, which is under tight regulatory control of ShvR, is essential for transition from intracellular persistence of *Burkholderia cenocepacia* to acute pro-inflammatory infection

**DOI:** 10.1371/journal.ppat.1007473

**Published:** 2018-12-04

**Authors:** Margarida C. Gomes, Yara Tasrini, Sujatha Subramoni, Kirsty Agnoli, Joana R. Feliciano, Leo Eberl, Pamela Sokol, David O’Callaghan, Annette C. Vergunst

**Affiliations:** 1 VBMI, INSERM, Université de Montpellier, Nîmes, France; 2 Department of Microbiology, Immunology, and Infectious Diseases, University of Calgary, Calgary, Canada; 3 Department of Plant and Microbial Biology, University of Zürich, Zürich, Switzerland; Channing Laboratory, Brigham and Women's Hospital, UNITED STATES

## Abstract

The opportunistic pathogen *Burkholderia cenocepacia* is particularly life-threatening for cystic fibrosis (CF) patients. Chronic lung infections with these bacteria can rapidly develop into fatal pulmonary necrosis and septicaemia. We have recently shown that macrophages are a critical site for replication of *B*. *cenocepacia* K56-2 and the induction of fatal pro-inflammatory responses using a zebrafish infection model. Here, we show that ShvR, a LysR-type transcriptional regulator that is important for biofilm formation, rough colony morphotype and inflammation in a rat lung infection model, is also required for the induction of fatal pro-inflammatory responses in zebrafish larvae. ShvR was not essential, however, for bacterial survival and replication in macrophages. Temporal, rhamnose-induced restoration of *shvR* expression in the *shvR* mutant during intramacrophage stages unequivocally demonstrated a key role for ShvR in transition from intracellular persistence to acute fatal pro-inflammatory disease. ShvR has been previously shown to tightly control the expression of the adjacent *afc* gene cluster, which specifies the synthesis of a lipopeptide with antifungal activity. Mutation of *afcE*, encoding an acyl-CoA dehydrogenase, has been shown to give similar phenotypes as the *shvR* mutant. We found that, like *shvR*, *afcE* is also critical for the switch from intracellular persistence to fatal infection in zebrafish. The closely related *B*. *cenocepacia* H111 has been shown to be less virulent than K56-2 in several infection models, including *Galleria mellonella* and rats. Interestingly, constitutive expression of *shvR* in H111 increased virulence in zebrafish larvae to almost K56-2 levels in a manner that absolutely required *afc*. These data confirm a critical role for *afc* in acute virulence caused by *B*. *cenocepacia* that depends on strain-specific regulatory control by ShvR. We propose that ShvR and AFC are important virulence factors of the more virulent Bcc species, either through pro-inflammatory effects of the lipopeptide AFC, or through AFC-dependent membrane properties.

## Author summary

*Burkholderia cenocepacia* is a bacterial pathogen that can infect cystic fibrosis patients, causing a chronic infection that can suddenly change to often fatal inflammatory necrotic pneumonia called cepacia syndrome. The transcriptional regulator ShvR controls the expression of a lipoprotein with antifungal properties (AFC). Using zebrafish larvae, we earlier found that macrophages provided an important replication niche for these bacteria, and were required for the strong pro-inflammatory response. Here, we contribute new data that show that ShvR and AfcE are absolutely required for the induction of pro-inflammatory disease by *B*. *cenocepacia*. In the absence of ShvR and AfcE *B*. *cenocepacia* is restricted to macrophages. Using a sugar-dependent expression system that allowed us to turn on *shvR* or *afcE* expression in bacteria that lack *shvR* or *afc*, respectively, we were able to mimic the transition from a restricted intracellular stage to acute disseminated infection. This result supports an important role for ShvR/AFC in the acute virulence of *B*. *cenocepacia*.

## Introduction

*B*. *cenocepacia* belongs to the *Burkholderia cepacia* complex (Bcc), currently encompassing 21 officially named species [1,2]. These opportunistic bacteria are notorious pathogens of cystic fibrosis (CF) patients [3,4] and are emerging as the culprit of serious infections in non-CF conditions, both in and outside the hospital [5–8]. Infection of CF airways by bacteria belonging to the Bcc can be asymptomatic but often result in chronic infection with intermittent acute exacerbations resulting in progressive worsening of lung function [9,10]. *B*. *cenocepacia* is particularly associated with reduced survival and a high risk for development of unpredictable acute fatal necrotizing pneumonia and sepsis, termed “cepacia syndrome”. Bcc bacteria are well-known for their intrinsic resistance to stress conditions and antibiotics [11–13], complicating disease management and treatment strategies.

Bcc bacteria are ubiquitously present in the environment and in industrial settings; they have evolved intricate signalling networks to rapidly adapt to changing or stressful environments, for instance in competition for nutrients with other bacteria or fungi, or as a defence mechanism against nematodes and protozoan hosts [14–18]. These signalling networks could also play important roles in pathogenicity and adaptation during human opportunistic infections. As an example, the GacA/GacS signalling cascade in *Pseudomonas* spp. has been shown to be involved in adaptation to natural conditions, while playing a crucial role during infection of CF patients by *P*. *aeruginosa* by providing a switch from acute (Type 3 secretion system-dependent) to chronic (biofilm life stage) infections [19,20].

Several regulators, including the quorum sensing systems CepI/R and CciI/R, and the global regulator AtsR, have been shown to be involved in virulence of *B*. *cenocepacia* using different model hosts [21–23]. ShvR is a LysR-type transcriptional regulator (LTTR) that has been shown to be involved in lung inflammation caused by *B*. *cenocepacia* K56-2 in a rat agar bead model of chronic lung infection [24]. The *shvR* mutant, however, was highly persistent in the infected rat lungs. In addition, the absence of ShvR resulted in loss of antifungal activity, reduced biofilm formation on abiotic surfaces [24] and a loss of rough colony morphotype, hence the name “Regulator of SHiny Variant”. The absence of *shvR* has been shown to result in moderate changes in the expression of over 1000 genes [25]. Interestingly, the expression of the adjacent *afc* operon, encoding 24 proteins specifying the synthesis of a lipopeptide with antifungal activity against *Rhizoctonia solani* [26] was reduced 100-fold, showing it is under tight positive regulatory control of ShvR [25]. Mutation of the *afcE* gene (BCAS0208), encoding an acyl-CoA dehydrogenase, resulted in the same phenotypes as those seen for the *shvR* mutant, including loss of antifungal activity, reduced biofilm formation, and shiny colony morphology [25,27,28]. Furthermore, mutation of *afcE* resulted in significantly reduced chronic lung inflammation in experimentally infected rats, similar to that found for a *shvR* mutant [28]. The high persistence of the *shvR* mutant in a rat infection model, alongside its reduced ability to form biofilms prompted us to investigate a role for ShvR and AfcE in virulence of *B*. *cenocepacia* K56-2 using zebrafish embryos, particularly their contribution during intramacrophage stages.

*B*. *cenocepacia* is an intracellular pathogen and we have recently shown that macrophages play a crucial role in bacterial proliferation and acute fatal infection by *B*. *cenocepacia in vivo* using zebrafish larvae [29]. Importantly, other Bcc strains were able to persist in macrophages, but could not efficiently disseminate and induce robust host pro-inflammatory responses [29,30]. The reason for these different disease outcomes, which may have important clinical impact, is not clear. In this work, we find that the expression of *shvR* and *afcE* is critical for *B*. *cenocepacia* K56-2 to progress from an intracellular persistent stage with low pro-inflammatory responses, to disseminated acute fatal infection. Bioinformatic analysis indicates that the *shvR/afc* genes are present in the genomes of 50% of the 19 Bcc species sequenced to date. *B*. *cenocepacia* H111 is closely related to K56-2, but shows reduced virulence in different animal models, including zebrafish. We show that acute virulence of H111 also depends on the presence of the *afc* cluster. Strikingly, constitutive expression of *shvR* from a *lac*-promoter increased virulence of H111 to almost K56-2 levels in an *afc*-dependent manner. Altogether, our results demonstrate an important role for the *afc* gene cluster in pathogenicity, the severity of which may depend on strain-specific upstream regulatory control mechanisms.

## Results

### A *shvR* mutant is attenuated in virulence in zebrafish embryos

**Fig 1:**
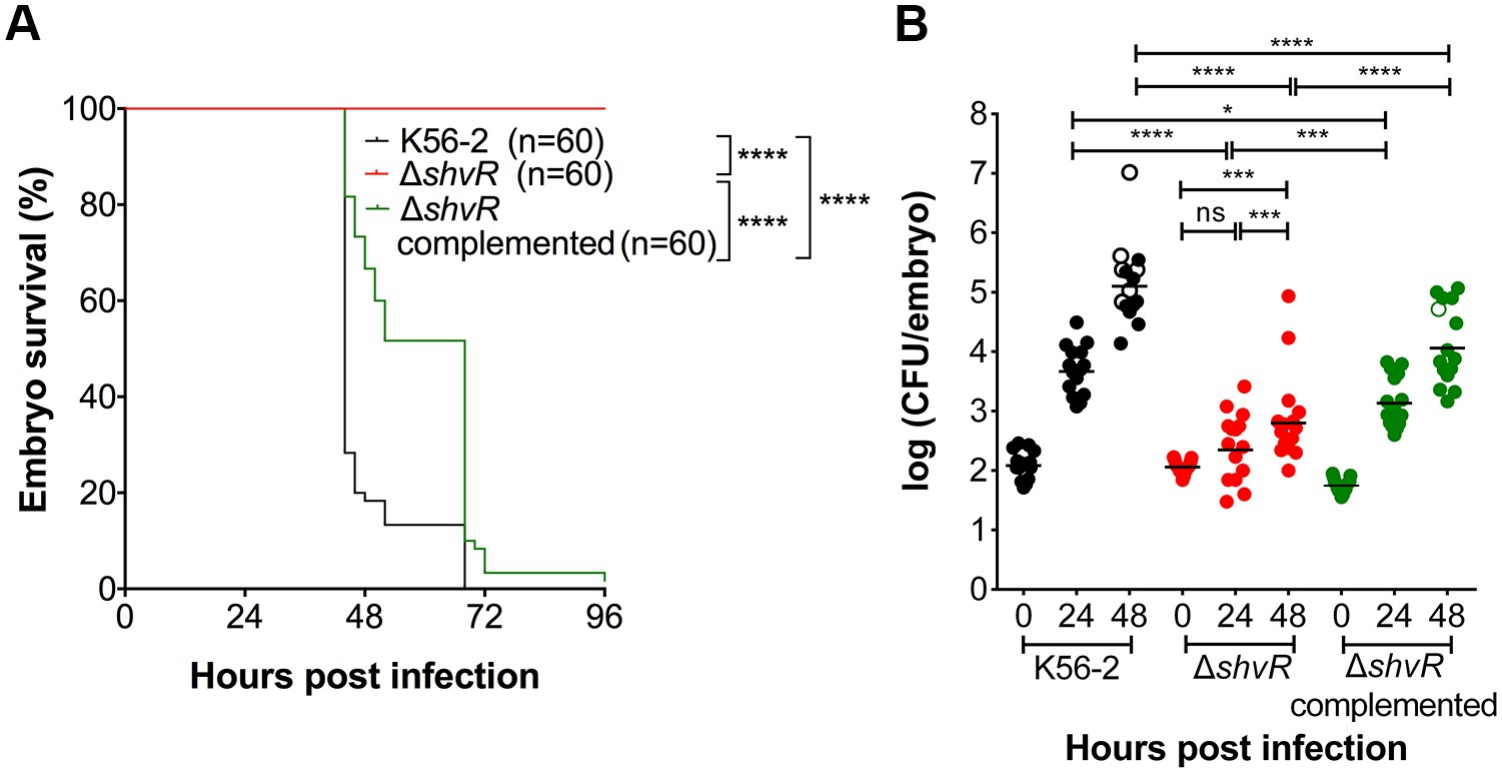
ShvR is important for fatal infection in zebrafish larvae. Zebrafish larvae were micro-injected with on average 143 ± 76 CFU of wildtype *B*. *cenocepacia* K56-2 (black), 118 ± 29 CFU of Δ*shvR* (red), and 58 ± 16 CFU of complemented Δ*shvR* (green), and embryo survival (A) and bacterial burden (B) were determined. The complemented strain harboured a low copy pCR11-derivative expressing *shvR* from its endogenous promoter. Open circles indicate dead embryos. Pooled results from three independent experiments are shown (n = 20 (A) and n = 5 (B), respectively, per group per experiment). Geometric means with each data point representing an individual embryo. A log rank (Mantel-Cox) test was used to determine significance of survival differences (A). Significance between groups was determined using one-way ANOVA with Sidak’s Multiple Comparisons test (B). * p ≤ 0.05, ** p ≤ 0.01, *** p ≤ 0.001, **** p ≤ 0.0001, ns: not significant.

We first analysed the role of *B*. *cenocepacia* K56-2 ShvR in virulence in the zebrafish infection model. Zebrafish embryos were micro-injected at 30 hours post-fertilization (hpf) with *B*. *cenocepacia* K56-2(mCherry_pCR11_), Δ*shvR*(mCherry_pCR11_) or the complemented Δ*shvR*(p*shvR*:*shvR*;mCherry_pCR11_). Analysis of embryo survival showed that Δ*shvR* was unable to cause fatal infection over the time course of the experiment, in contrast to its wildtype parent which caused 100% embryo mortality in 3 days (Fig 1A and [30]). In agreement with the observed reduced host mortality there was a significantly lower bacterial burden of Δ*shvR* compared to K56-2 at 24 and 48 hours post infection (hpi) (Fig 1B). Although all embryos survived infection with Δ*shvR*, some contained significantly more bacteria at 24 and 48 hpi compared to T = 0 (Fig 1B), which suggests the mutant bacteria are able to replicate. Expression of *shvR* from its endogenous promoter introduced on a single copy plasmid restored virulence to the Δ*shvR* mutant (Fig 1). These results show that ShvR is required for development of acute fatal infection.

### *ShvR* mutant bacteria persist and replicate in macrophages

**Fig 2:**
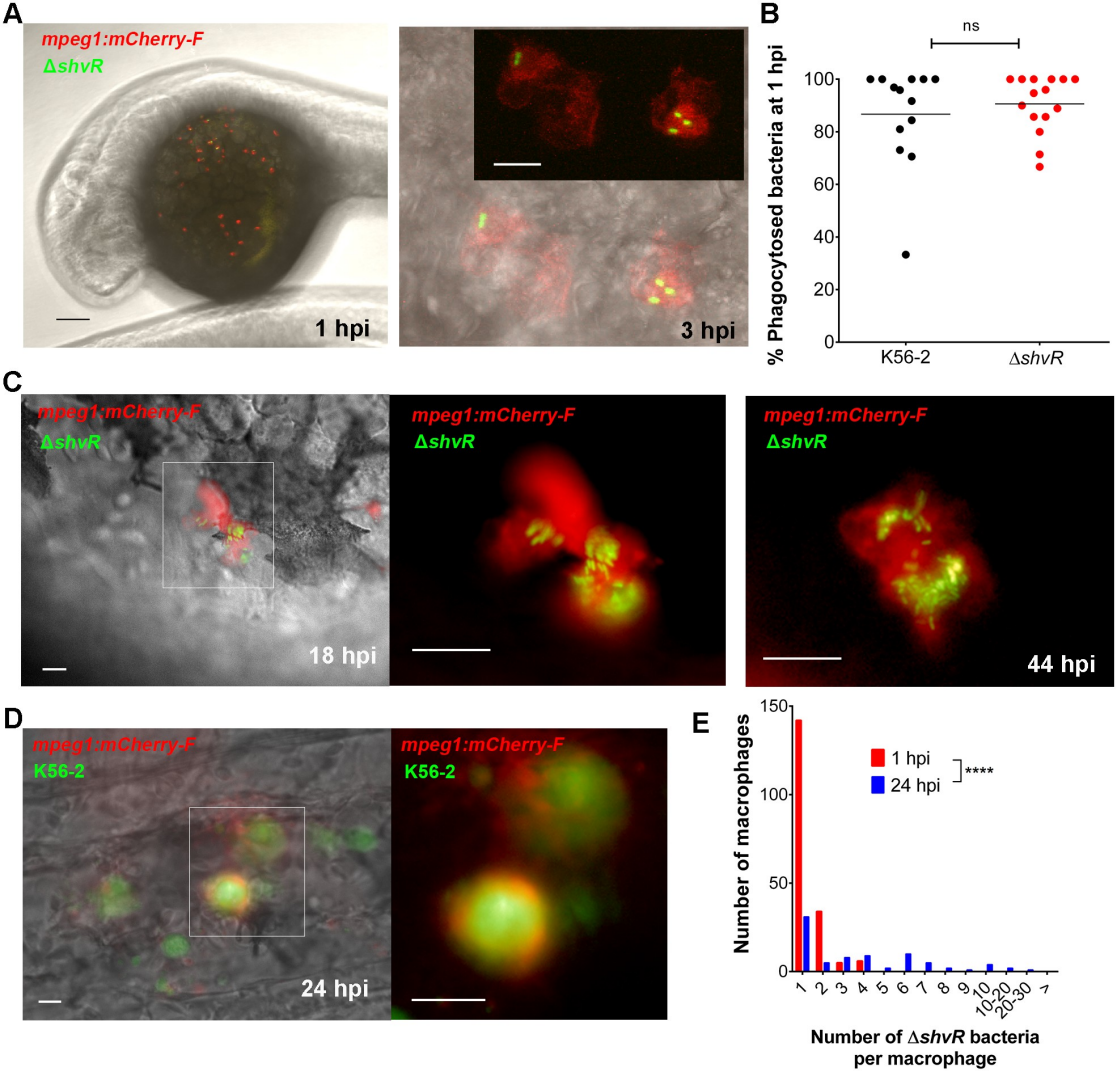
Δ*shvR* mutant bacteria are able to persist and replicate in macrophages. **(A)** Tg(*mpeg1*:*mCherry-F*) embryos were injected intravenously at 30 h post fertilization with ~50 CFU Δ*shvR* bacteria expressing eGFP (pIN301). Left panel: Confocal stack image over the yolk region of a representative embryo (macrophages marked in red) at 1 hpi. Scale bar 100 μm. Right panel: Detailed confocal (bright field and fluorescent overlay) image stack of two macrophages at 3 hpi containing 2 and 3 Δ*shvR* bacteria, respectively. Inset shows red and green channels only. Scale bar 10 μm. S1 Movie shows a video of the individual (21) sequential images. **(B)** Quantification of intracellular and extracellular bacteria in PFA-fixed embryos at 1 hpi over the yolk as indicated in Fig 2A using confocal microscopy. Each data point represents an individual Tg(*mpeg1*:*mCherry-F*) transgenic embryo injected intravenously with either wildtype *B*. *cenocepacia* K56-2 (black dots, n = 13) or Δ*shvR* (red dots, n = 15) expressing eGFP. Counts per embryo are presented as percentage of internalized bacteria relative to total numbers of bacteria in the yolk sac region. A total of 220 and 141 bacteria were counted for K56-2 and Δ*shvR*, respectively. Unpaired t-test, with mean 86.7 ± 5.3 and 90.6 ± 2.8, respectively, p-value 0.51. Data in (A, B) are representative of two independent experiments. **(C)** Tg(*mpeg1*:*mCherry-F*) embryos were injected with Δ*shvR* expressing eGFP. Images of an infected macrophage at 18 hpi over the yolk (left, bright field and fluorescent overlay image; enlarged inset, red and green overlay) and at 44 hpi (red and green overlay image) with high numbers of intracellular bacteria (representative of at least 5 embryos per treatment in more than 3 experiments). Scale bars, 12.5 μm. **(D)** Tg(*mpeg1*:*mCherry-F*) embryos were injected with K56-2 wildtype expressing eGFP. Image of an infection site at 24 hpi in the tail region (left, bright field and fluorescent overlay image; enlarged inset, red and green overlay) with high numbers of bacteria. Scale bars, 12.5 μm. **(E)** Quantification of the number of intracellular Δ*shvR* bacteria per macrophage in PFA-fixed embryos at 1 hpi (mean: 1.3 ± 0.05 bacteria per macrophage) and 24 hpi (4.1 ± 0.47 SEM), presented as frequency distribution histogram. The graph represents a total of 187 (15 embryos) and 80 macrophages (10 embryos), at 1 and 24 hpi respectively. Mann Whitney test p < 0.0001. Representative of 2 experiments.

Our previous work showed that macrophages efficiently phagocytose intravenously injected *B*. *cenocepacia* K56-2, and that they are critical for bacterial replication and ensuing fatal pro-inflammatory infection in zebrafish embryos [29]. Phagocytosis of bacteria from the blood results in infected macrophages distributed over the yolk and the tail region [30]. To analyse the interaction of Δ*shvR* with macrophages in the host, we quantified the percentage of Δ*shvR* bacteria that were internalised by macrophages using confocal imaging of infected Tg(*mpeg*:*mCherry-F*) transgenic embryos, which express a membrane localised mCherry protein (Fig 2A and S1 Movie). One hour after injection, Δ*shvR* bacteria were as efficiently phagocytosed by macrophages as wildtype bacteria (Fig 2B). Real time experiments showed that the Δ*shvR* bacteria still localised in macrophages at later time points, where they sometimes reached high numbers (Fig 2C) in agreement with the observed increase in CFU counts (Fig 1B). In contrast, at these later time points wildtype K56-2 bacteria have replicated to very high intracellular numbers, formed local infection sites (Fig 2D), re-entered the blood circulation and spread systemically as shown earlier [29]. Quantification of the number of intracellular Δ*shvR* bacteria in infected macrophages using Tg(*mpeg*:*mCherry-F*) embryos at 1 hpi and 24 hpi (Fig 2E) showed a significant decrease in the number of macrophages containing 1 or 2 bacteria and an increase in the number of macrophages containing more than 4 bacteria, respectively, demonstrating that the Δ*shvR* mutant is able to survive and replicate inside macrophages. The total number of infected macrophages that were observed per embryo decreased in time from 12.3 ± 2.1 (SEM; n = 15 embryos) at 1 hpi to 7.6 ± 0.9 (SEM; n = 10 embryos) at 24 hpi, suggesting that a proportion of Δ*shvR* bacteria can establish a replication niche, while others get degraded.

**Fig 3:**
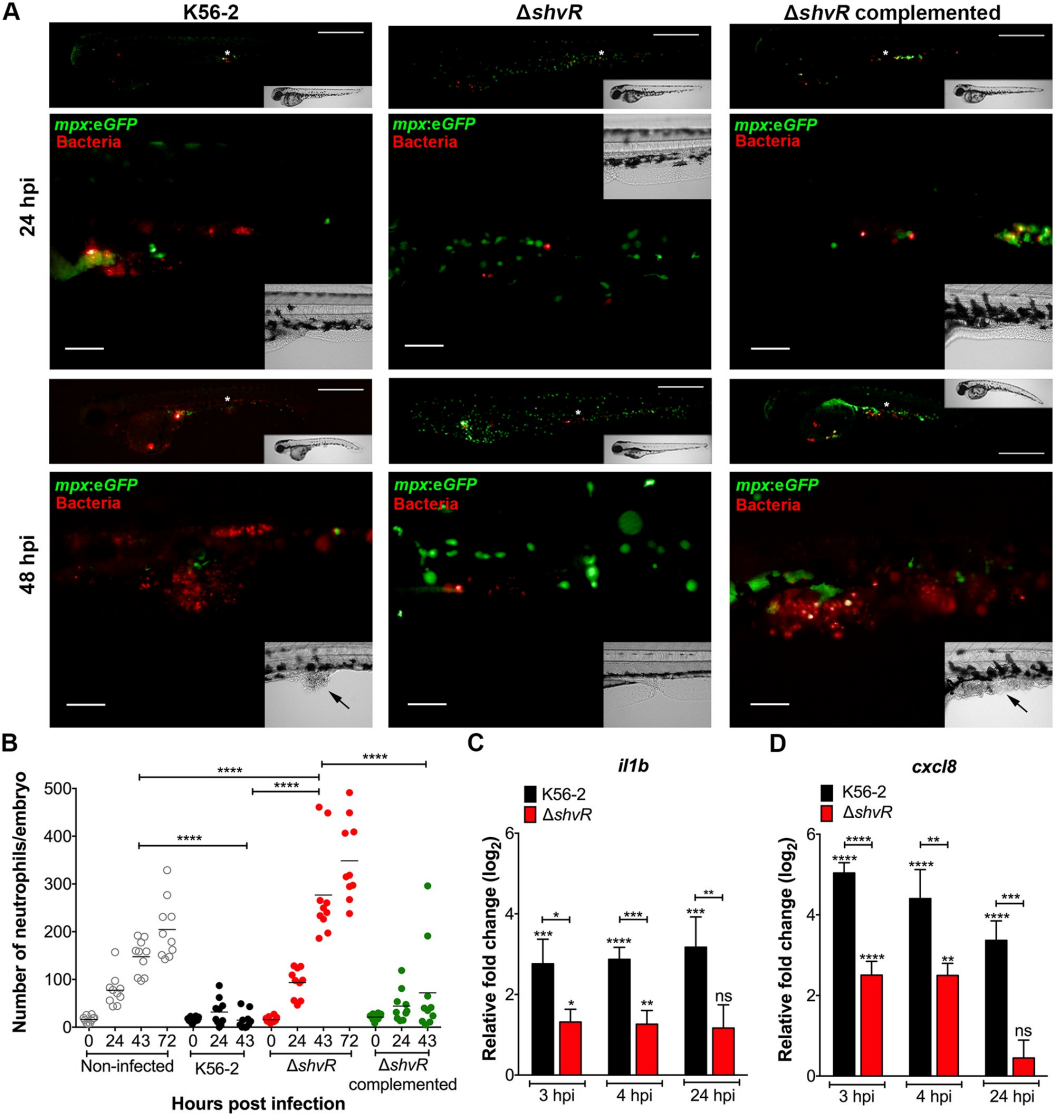
A Δ*shvR* mutant is unable to spread and elicit strong pro-inflammatory responses. *B*. *cenocepacia* K56-2, Δ*shvR*, and complemented Δ*shvR* were microinjected intravenously in embryos at 30 hpf. **(A)** Real time imaging of representative Tg(*mpx*:e*GFP*) injected larvae (neutrophils green) with fluorescent bacteria (red filter) at 24 and 48 hpi. For each bacterial strain, the same embryo was imaged at both time points. White asterisks in upper images indicate the region imaged with a higher magnification objective and presented underneath. Black arrows in bright field inset indicate tissue damage. Scale bar, 50 μm. **(B)** Mean neutrophil numbers in Tg(*mpx*:e*GFP*) larvae of non-infected control (grey open circles) and infected with K56-2 (black circles), Δ*shvR* (red circles) and complemented mutant (green circles). Representative images of each group, and an example of a binary conversion image used for pixel quantification are provided in S1 Fig. Quantification was performed prior to injection (0 hpi) and at 24 and 48 hpi. Only in non-infected and Δ*shvR*-infected embryos the 72 hpi time points were considered as embryos were still alive. Geometric means with each data point representing an individual embryo. Pooled results from two independent experiments are shown (n = 5 respectively per group per experiment). Significance was determined using one-way ANOVA with Sidak’s Multiple Comparisons test. **(C, D)** Mean relative *il1b* and *cxcl8* gene expression levels (qRT-PCR) in embryos injected with on average 273 ± 114 CFU of *B*. *cenocepacia* K56-2 (black bars) or 184 ± 111 CFU of Δ*shvR* (red bars). Normalization was performed to the PBS-injected control group at each time point. Asterisks above each bar indicate significance compared to the PBS control at each time point, and significance between groups per time point is indicated with a horizontal line. Error bars represent mean with SEM of three independent experiments. Statistical analysis of qRT-PCR data was performed using one-way ANOVA with Tukey’s Multiple Comparison Test. **(B, C, D)** * p ≤ 0.05, ** p ≤ 0.01, *** p ≤ 0.001, **** p ≤ 0.0001, ns: not significant.

In contrast to wildtype K56-2, which disseminates from initially infected macrophages at around 10–12 hpi culminating in acute fatal infection [29,30], Δ*shvR* did not spread throughout the embryos. Instead, the infection was characterised by low overall bacterial burden and the absence of tissue inflammation as seen for K56-2 and the complemented mutant (Fig 3A). However, real time observations showed that small infection sites with multiple infected cells sometimes developed from single infected macrophages. Although we have been unable to quantify this event due to the cell dynamics of the *in vivo* infection with immune cells moving around, and the mechanism behind the local spread is not known, it suggests the Δ*shvR* mutant can maintain low levels of chronic infection. Together, the results demonstrate that ShvR is not essential for intracellular persistence and replication, but that it regulates factors that are critical for the bacteria to disseminate efficiently and cause robust pro-inflammatory infection.

### The innate immune response towards the *shvR* mutant is significantly reduced compared to wildtype

We have shown earlier that acute infection caused by *B*. *cenocepacia* K56-2 correlates with systemic phagocyte death, whereas persistent infection, caused by for instance *B*. *stabilis* LMG14294, triggers neutrophilic inflammation [29]. Neutrophil numbers were quantified during infection with wildtype K56-2, Δ*shvR* and the complemented mutant at 0, 24, 43, and 72 hpi using Tg(*mpx*:*eGFP*) transgenic embryos, which express GFP specifically in neutrophils (S1 Fig and Fig 3B). Embryos infected with *B*. *cenocepacia* K56-2 showed a strong decline in the number of neutrophils, with only few cells remaining at the end stage of infection (Fig 3B and [29]). In contrast, infection with Δ*shvR* resulted in neutrophilic inflammation with a significant increase in the number of neutrophils compared to non-infected control embryos from 43 hpi (Fig 3B). The complemented mutant caused neutropenia, as seen for the wildtype strain, confirming restoration of virulence by expression of *shvR in trans*.

*B*. *cenocepacia* K56-2 induces a robust increase in host pro-inflammatory cytokine expression ([29]; Fig 3C and 3D). The expression of *il1b* and *cxcl8*/*il8* upon infection with Δ*shvR* was, however, only moderately increased compared to PBS-injected embryos (Fig 3C and 3D), as shown earlier for *B*. *stabilis* [29]. Moreover, at 24 hpi, no significant difference in gene expression was observed between embryos injected with PBS or Δ*shvR*. These results confirm that ShvR is required for the induction of robust pro-inflammatory responses.

### ShvR tightly regulates factors required for transition from intramacrophage stages to acute fatal infection

**Fig 4:**
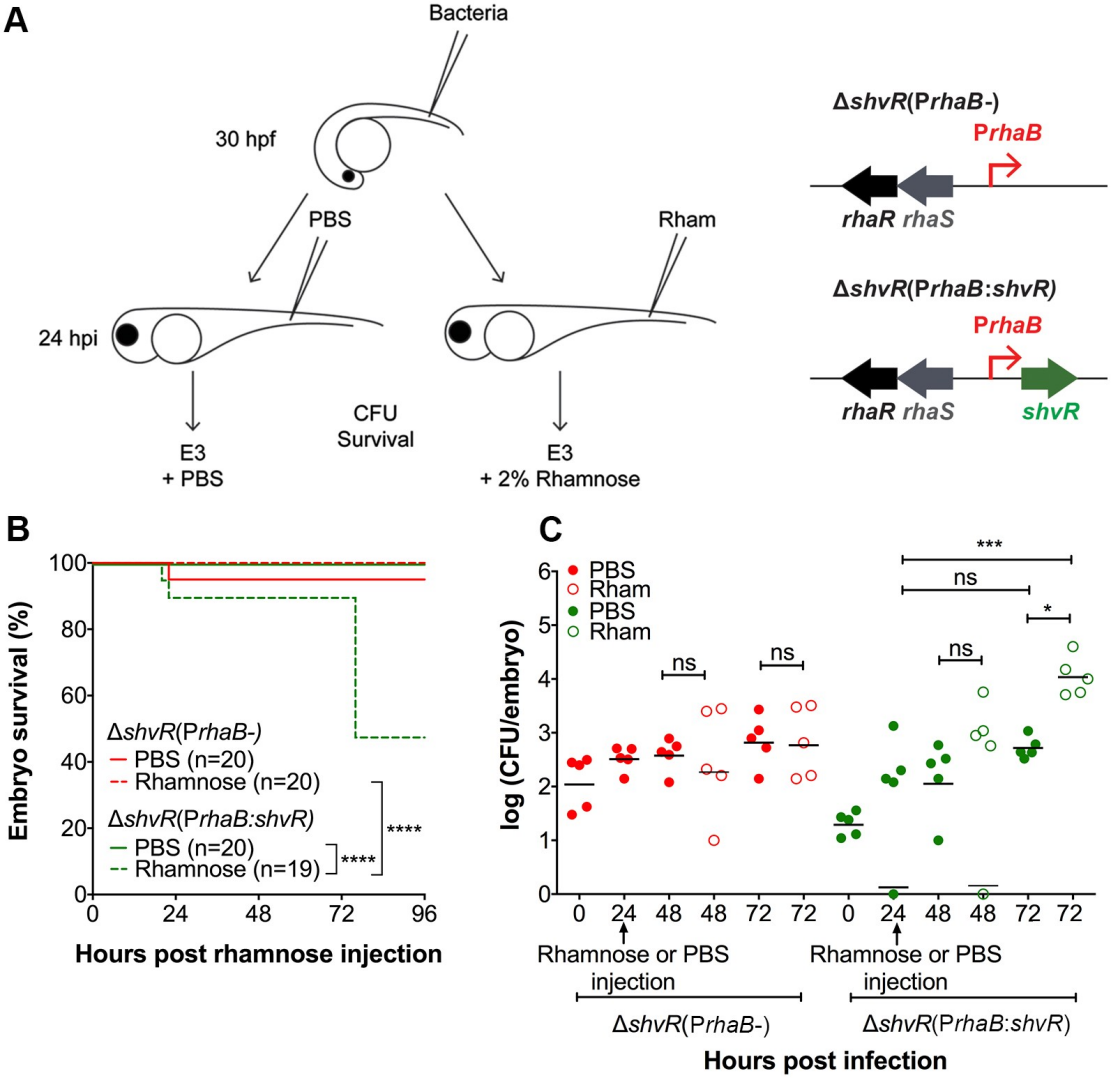
ShvR tightly controls bacterial factors needed for fatal infection. **(A)** Schematic representation of rhamnose-inducible *shvR* (P*rhaB*:*shvR*) and control (P*rhaB*-) constructs, cloned on a pBBR replication unit transferred into Δ*shvR* (right panel). Both strains were injected intravenously at 30 hpf. Zebrafish embryos were incubated for 24 hours in E3 water. At 24 hpi, PBS or 2% rhamnose, respectively, were injected intravenously to induce *shvR* gene expression (left panel). **(B, C)** Embryo survival and CFU counts after injection of 183 ± 136 CFU of *B*. *cenocepacia* K56-2 Δ*shvR*(P*rhaB*-) (red) and 22 ± 10 CFU of Δ*shvR*(P*rhaB*:*shvR*) (green), followed by injection of rhamnose or PBS, respectively at 24 hpi (black arrows). A representative experiment of three is shown (n = 20 (B) and n = 5 (C) respectively per group). **(B)** Solid lines indicate PBS-injected embryos while dashed lines indicate rhamnose -injected embryos. Differences in survival were determined using a Log rank (Mantel-Cox) test. **(C)** Bacterial burden was analysed at 0, 24, 48 and 72 hpi. The determination of CFU at 24 hpi was performed prior to rhamnose or PBS injection. Solid circles indicate PBS-injected embryos and open circles rhamnose injected embryos. Geometric means with each data point representing an individual embryo. Significance between groups was determined using one-way ANOVA with Sidak’s Multiple Comparisons test. * p ≤ 0.05, ** p ≤ 0.01, *** p ≤ 0.001, **** p ≤ 0.0001, ns: not significant.

Based on the requirement for macrophages for intracellular bacterial replication of K56-2 and subsequent pro-inflammatory fatal infection [29], and the restriction of the Δ*shvR* mutant to macrophages, we hypothesised that ShvR could be an important switch between intracellular persistence and acute pro-inflammatory infection. To address this hypothesis, we used a rhamnose inducible expression system [31] to temporally control the induction of *shvR* expression in the Δ*shvR* mutant during infection in zebrafish embryos. This allowed us to study the effect of experimental induction of *shvR* gene expression during intramacrophage stages of *B*. *cenocepacia* on disease outcome. The *shvR* coding sequence was cloned under control of the rhamnose-inducible promoter P*rhaB*, resulting in P*rhaB*:*shvR* and introduced in Δ*shvR*. The P*rhaB*- control plasmid, lacking the *shvR* coding sequence, served as a negative control. Zebrafish embryos were injected at 30 hours post fertilisation (hpf) with Δ*shvR*(P*rhaB*-) and Δ*shvR*(P*rhaB*:*shvR*). At 24 h post injection of bacteria, at the time Δ*shvR* resided in macrophages (Fig 2), a 2% rhamnose solution, or PBS as a control, was injected into the circulation of the infected embryos (see Fig 4A for schematic representation). In order to maintain *shvR* expression, the embryos were further incubated in E3 medium with 2% rhamnose. The presence of P*rhaB*- in Δ*shvR* had no effect on embryo survival or bacterial burden, even after injection of rhamnose (Fig 4B and 4C). Strikingly, application of rhamnose, but not PBS, restored virulence to Δ*shvR* harbouring P*rhaB*:*shvR*, measured as host mortality and increase in bacterial burden (Fig 4B and 4C). These results confirm a critical role for ShvR in acute infection.

**Fig 5:**
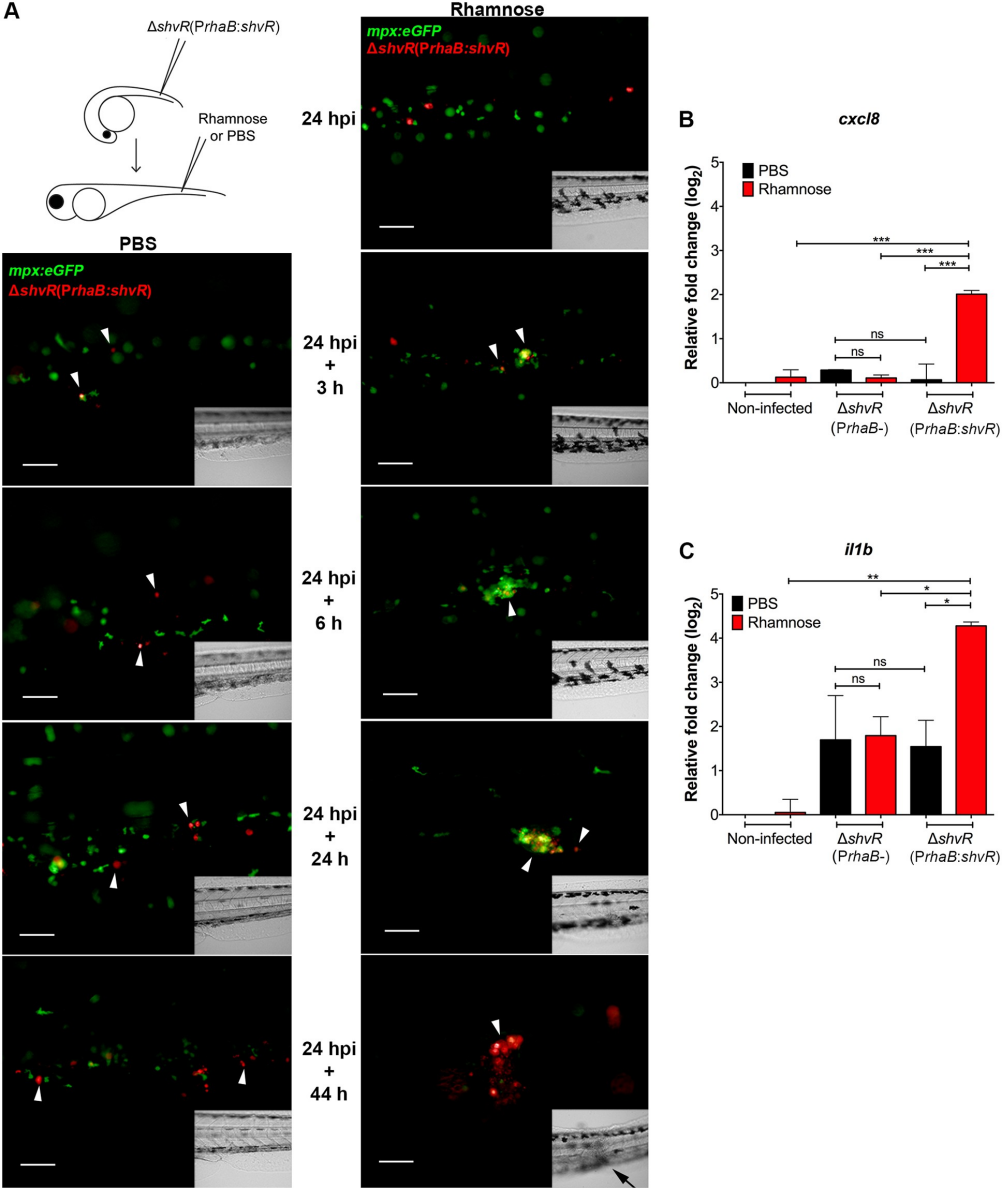
ShvR-mediated transition from persistent to acute pro-inflammatory infection. **(A)** Real time imaging of Tg(*mpx*:e*GFP*) embryos (green neutrophils) injected with Δ*shvR*(P*rhaB*:*shvR*) (in red), without (PBS) and with induction of *shvR* gene expression (rhamnose) at 24 hpi. Fluorescence imaging was performed at 0, 3, 6, 24 and 44 h post PBS or rhamnose injection. A single embryo was followed and visualised in time for each of the two conditions (representative embryos of a total of 20 embryos per experiment per condition are shown, 3 experiments). Green and red overlay images are shown. Inset shows the same image in bright field. White arrow heads indicate cells full of bacteria. Black arrow in bright field inset indicates tissue damage. Scale bars, 50 μm **(B, C)** Mean relative *il1b* and *cxcl8* expression levels analysed by qRT-PCR at 24 h post injection with rhamnose in non-infected and infected embryos. Embryos were injected with an average of 102 ± 37 CFU of *B*. *cenocepacia* K56-2 Δ*shvR*(P*rhaB*-) and an average of 74 ± 45 CFU of Δ*shvR*(P*rhaB*:*shvR*). At 24 hpi embryos were injected with either PBS or rhamnose. Black bars indicate PBS-injected embryos and red bars represent rhamnose-injected embryos. Normalization was performed to a PBS-injected non-infected control group. Injection of rhamnose had no significant effect on *il1b* and *cxcl8* expression (non-infected rhamnose-injected control). Significance between groups per time point is indicated with a horizontal line. Error bars represent mean with SEM of two independent experiments. Statistical analysis was performed using one-way ANOVA with Tukey’s Multiple Comparison Test. * p ≤ 0.05, ** p ≤ 0.01, *** p ≤ 0.001, **** p ≤ 0.0001, ns: not significant.

To visualise the effect of temporal induction of *shvR* expression on intracellular *B*. *cenocepacia*, fluorescence microscopy was performed using Tg(*mpx*:*eGFP*) transgenic embryos infected with Δ*shvR*(P*rhaB*:*shvR*) expressing mCherry. While injection with PBS in embryos infected prior with Δ*shvR*(P*rhaB*:*shvR*) did not alter the infection progression of the Δ*shvR* mutant, injection of rhamnose resulted in changes that are characteristic of acute infection (Fig 5A). These changes include neutrophil recruitment towards infection sites, indicative of increased pro-inflammatory signalling, and as described for acute infection with *B*. *cenocepacia* K56-2 [29]. The infection rapidly worsened and at 24 h post rhamnose injection the embryos were neutropenic (Fig 5A), as seen for infection with wildtype (Fig 3A). The 2-log increase in bacterial burden within 48 hours post rhamnose injection (Fig 4C) was in agreement with real time observations using fluorescence microscopy and time lapse confocal imaging (Fig 5A and S2 Movie). At later time points after rhamnose injection, tissue damage as a sign of strong pro-inflammatory responses could be observed (Fig 5A).

Next, we analysed whether global cytokine gene expression levels were induced after restoration of *shvR* expression in Δ*shvR*(P*rhaB*:*shvR*) bacteria. While no difference in relative expression levels was observed between rhamnose and PBS conditions in embryos infected with Δ*shvR*(P*rhaB*-), a significant increase in the expression of *cxcl8* and *il1b* was observed in embryos infected with Δ*shvR*(P*rhaB*:*shvR*) after injection with rhamnose (Fig 5B and 5C). Thus, ShvR strongly controls the expression of genes that are required for the transition from intracellular persistence to acute pro-inflammatory infection.

### *B*. *cenocepacia* K56-2 *afcE* determines the difference between intracellular persistence and acute fatal infection

ShvR has been described as a global regulator of virulence factors in *B*. *cenocepacia*, moderately (2 to 4 fold) changing the expression of a large set of genes. The antifungal activity cluster *afc*, comprising 24 genes organised in two divergently expressed operons localised adjacent to *shvR*, has been described to show strongly decreased expression levels (100 fold) in the absence of *shvR*, demonstrating *afc* expression is under tight positive control of ShvR [25]. In addition, mutation of *afcE* mimics the phenotypes found for Δ*shvR*, including shiny colony morphology, altered membrane properties, and reduced virulence in a rat infection model [25,27,28], suggesting the *afc* cluster is a major target of ShvR. In analogy to the experiments described above for Δ*shvR*, we therefore analysed a role for AfcE in persistent/acute transition.

**Fig 6:**
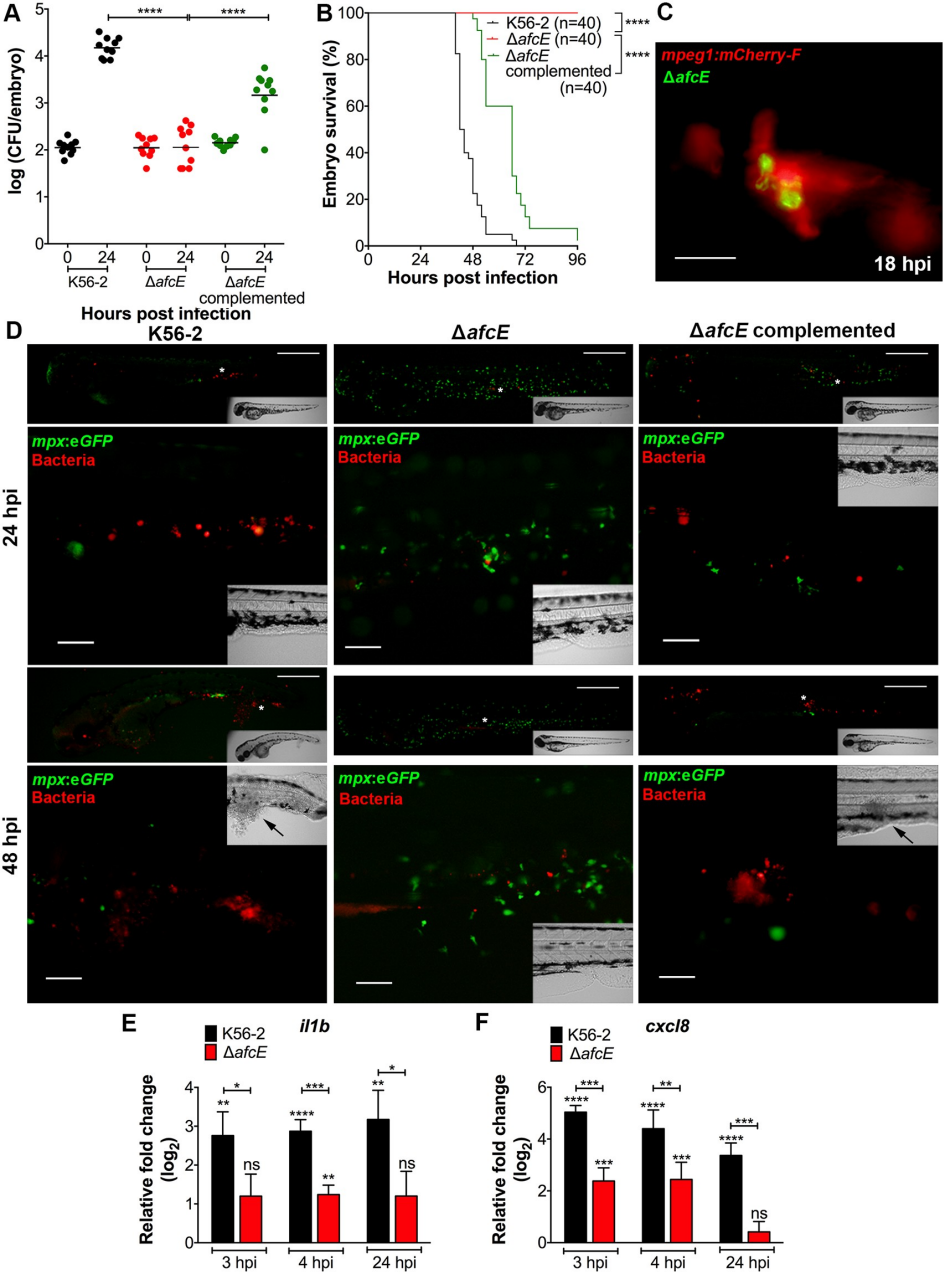
A Δ*afcE* mutant mimics the persistent infection caused by a Δ*shvR* mutant. Bacterial burden **(A)** and survival assays **(B)** of zebrafish embryos micro-injected with on average 120 ± 41 CFU of wildtype *B*. *cenocepacia* K56-2 (black), 126 ± 55 CFU of Δ*afcE* (red), and 146 ± 31 CFU of complemented Δ*afcE* (green) with P*lac*:*afcE*;DSRed_pBBR_ expressing *afc* from the *lac* promoter. Pooled results from two independent experiments are shown (n = 20 (A) and n = 5 (B) respectively per group per experiment). Geometric means with each data point representing an individual embryo. Significance between groups was determined using one-way ANOVA with Sidak’s Multiple Comparisons test (A). Statistical analysis of survival percentage was done a Log rank (Mantel-Cox) test (B). **(C)** Tg(*mpeg1*:*mCherry-F*) transgenic embryos, specifically expressing mCherry in macrophages, were injected with Δ*afcE* harbouring pIN301 (green). Representative image (red and green overlay) of an infected macrophage at 18 hpi. Scale bar, 12.5 μm. **(D)** Real time imaging of representative Tg(*mpx*:e*GFP*) injected larvae (neutrophils in green) with fluorescent bacteria (red) at 24 and 48 hpi. For each bacterial strain, the same embryo was imaged at both time points. White asterisks in upper images indicate the region imaged with a higher magnification objective and presented underneath. Black arrows in bright field inset indicate tissue damage. Scale bars, 50 μm. **(E, F)** Mean relative *il1b* and *cxcl8* gene expression levels (qRT-PCR) in embryos injected with on average 273 ± 114 CFU of *B*. *cenocepacia* K56-2 (black bars) or 101 ± 41 CFU of Δ*afcE* (red bars). Data for *afcE* were obtained in the same experiments shown in Fig 3, therefore the values for expression of the cytokines in K56-2 infection are the same in the graphs presented in both figures. Normalization was performed to PBS-injected non-infected control group at each time point. Asterisks above each bar indicate significance compared to the PBS control at each time point, and significance between groups per time point is indicated with a horizontal line. Error bars represent mean with SEM of three independent experiments. Statistical analysis was performed using one-way ANOVA with Tukey’s Multiple Comparison Test. **(A, B, E, F)** * p ≤ 0.05, ** p ≤ 0.01, *** p ≤ 0.001, **** p ≤ 0.0001, ns: not significant.

Infection experiments showed that Δ*afcE*(DSRed_pBBR_) was severely attenuated in virulence in zebrafish embryos (Fig 6A and 6B). In agreement with the inability of the mutant to cause fatal infection, Δ*afcE* showed significantly lower bacterial burden compared to K56-2 at 24 hpi, similar to that observed for Δ*shvR* (Fig 1). Expression of *afcE* in *trans* (Δ*afcE* (P*lac*:*afcE*;DSRed_pBBR_) complemented virulence to the Δ*afcE* mutant (Fig 6A and 6B). Δ*afcE* was taken up by macrophages as efficiently as the wildtype and Δ*shvR* mutant from the blood circulation (Fig 2B and S2A Fig). After phagocytosis by macrophages, Δ*afcE* bacteria were observed in macrophages also at later time points, sometimes, like Δ*shvR*, at high numbers (Fig 6C). Quantification of intracellular Δ*afcE* bacteria at 1 hpi and 24 hpi (S2B Fig) confirmed that the Δ*afcE* mutant is able to survive and replicate inside macrophages, as shown for Δ*shvR* (Fig 2E).

Using non-invasive real time imaging, we observed that the Δ*afcE* mutant bacteria did not cause neutropenia nor disseminated infection, while expression of the *afcE* gene restored these wildtype features (Fig 6D). Further, analysis of the global pro-inflammatory response of the host showed that the expression of the cytokine genes *cxcl8* and *il1b* is induced to lower levels during infection with Δ*afcE* compared to wildtype K56-2 (Fig 6E and 6F), similar to that shown for Δ*shvR* (Fig 3C and 3D). These results clearly demonstrate that AfcE is required for acute, disseminated pro-inflammatory infection.

**Fig 7:**
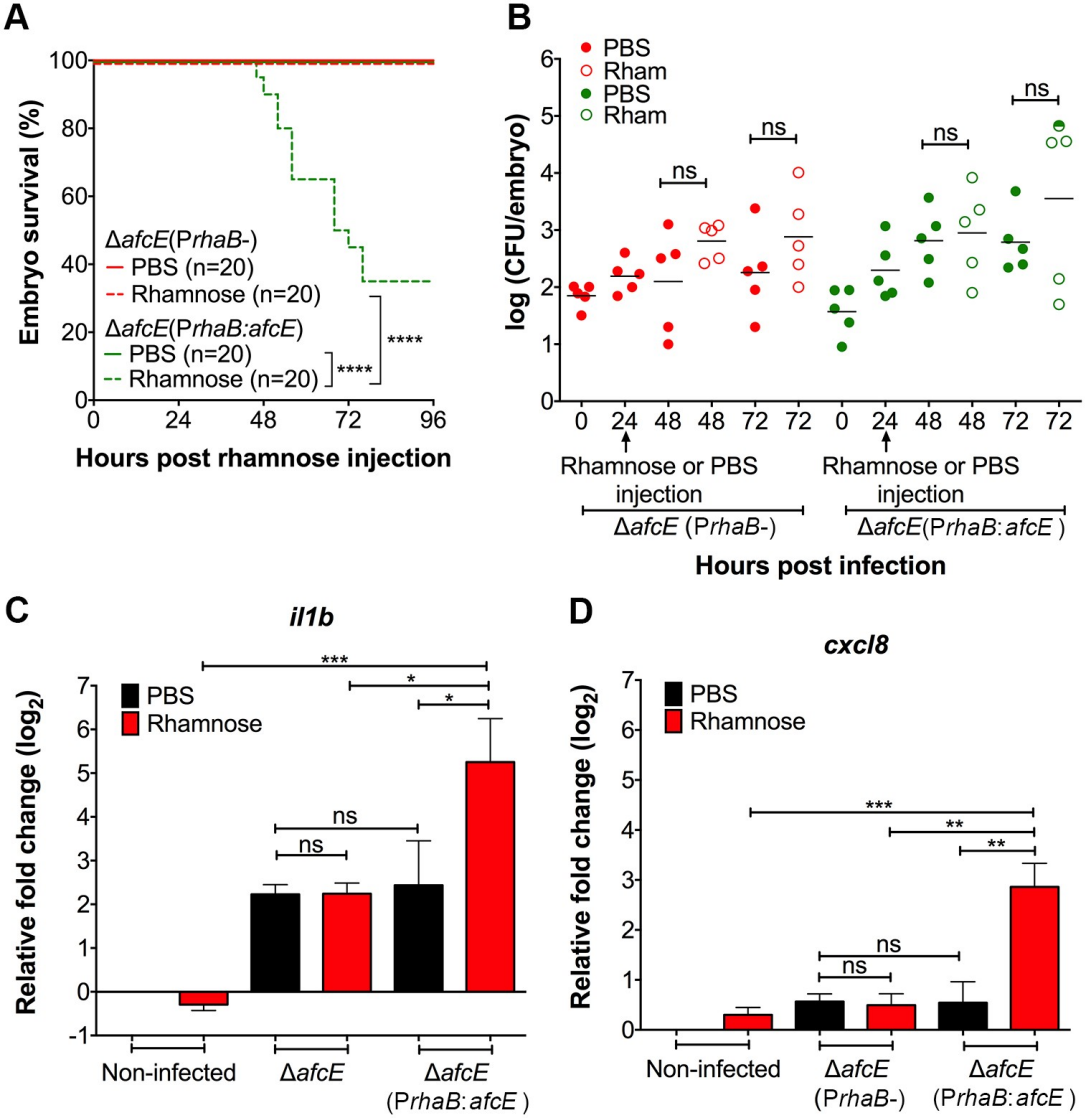
*AfcE* expression is critical for progression from persistent to disseminated acute fatal infection. Application of the experimental method depicted in Fig 4A using rhamnose inducible *afcE* (P*rhaB*:*afcE*) and control constructs (P*rhaB*-) introduced into Δ*afcE*. **(A,B)** Embryo survival and CFU counts upon injection of on average 76 ± 29 CFU of *B*. *cenocepacia* K56-2 Δ*afcE*(P*rhaB*-) (red) and 50 ± 37 CFU of Δ*afcE*(P*rhaB*:*afcE*) (green). A representative experiment of three is shown (n = 20 (A) and n = 5 (B) respectively per group). **(A)** Solid lines indicate PBS injected embryos and dashed lines rhamnose injected embryos. A Log rank (Mantel-Cox) test was used for statistical analysis. **(B)** Bacterial burden was analysed at 0, 24, 48 and 72 hpi. The determination of CFU at 24 hpi was performed before rhamnose or PBS injection. Closed circles indicate PBS-injected embryos and open circles rhamnose injected embryos. Semi-filled circle indicates dead embryo. Geometric means with each data point representing an individual embryo. Significance between groups was determined using one-way ANOVA with Sidak’s Multiple Comparisons test. **(C, D)** Mean relative *cxcl8* and *il1b* expression levels (qRT-PCR) at 24 h post injection of rhamnose. Embryos were injected with an average of 141 ± 42 CFU of Δ*afcE*(P*rhaB*-) and average 104 ± 45 CFU of Δ*afcE*(P*rhaB*:*afcE*). At 24 hpi embryos were injected with either PBS or rhamnose. Black bars indicate PBS-injected embryos and red bars represent rhamnose-injected embryos. Normalization was performed to PBS-injected non-infected control group. Significance between groups per time point is indicated with a horizontal line. Error bars represent mean with SEM of two independent experiments. Statistical analysis was done using one-way ANOVA with Tukey’s Multiple Comparison Test. * p ≤ 0.05, ** p ≤ 0.01, *** p ≤ 0.001, **** p ≤ 0.0001, ns: not significant.

Experiments using rhamnose-mediated induction of *afcE* during intramacrophage stages, as shown for *shvR* (Fig 4), were performed. Whereas infection with Δ*afcE*(P*rhaB*-) after injection of rhamnose or PBS at 24 hpi remained persistent, embryos infected with Δ*afcE*(P*rhaB*:*afcE*) and injected with rhamnose succumbed to the infection (Fig 7A), which correlated with an increase in bacterial CFU at 72 hpi (Fig 7B). Not all embryos showed increased bacterial numbers, however, and further experiments are needed to determine the reasons for the apparently larger biological variation in this assay for the Δ*afc*E mutant compared to the Δ*shvR* mutant (Fig 4C). In addition, global cytokine expression of embryos infected with Δ*afcE*(P*rhaB*:*afcE*) was significantly induced at 24 h post injection of rhamnose, but not PBS (Fig 7C and 7D). Together, these results demonstrate that AfcE is essential for bacterial dissemination and the robust pro-inflammatory fatal response seen for *B*. *cenocepacia* K56-2, in agreement with *afc* being the direct target of ShvR required for acute infection caused by *B*. *cenocepacia* K56-2.

### The *shvR-afc cluster* is present in a subset of the Bcc species

**Fig 8:**
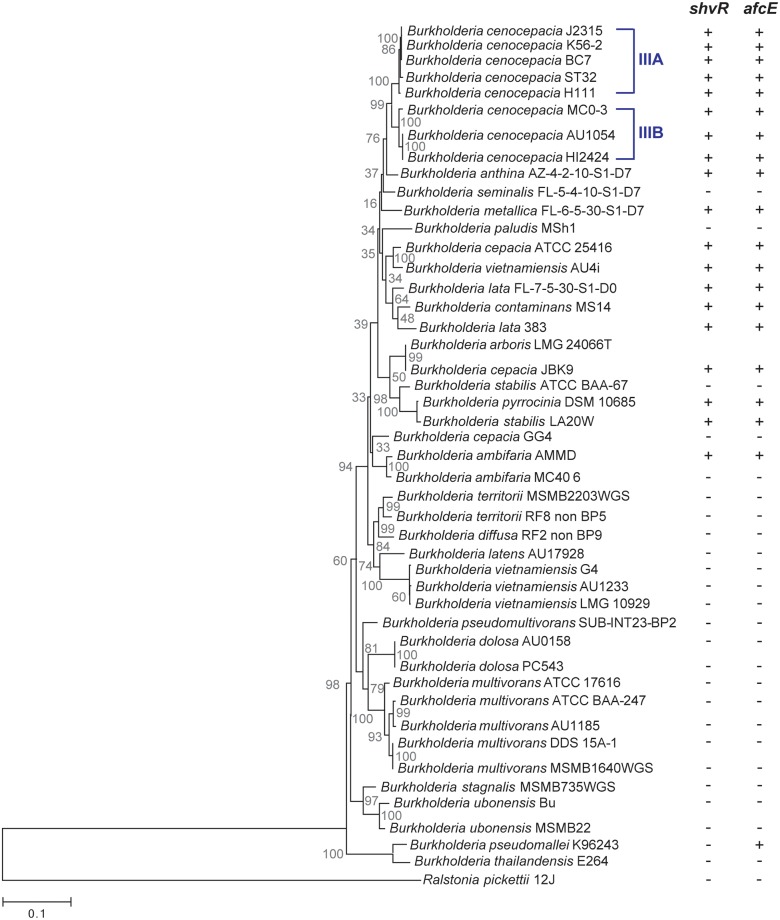
Phylogenetic inference of the *Burkholderia cepacia* complex species. Maximum likelihood phylogeny of 21 *Burkholderia cepacia* complex species (summarised in S2 Table) based on the alignment of concatenated nucleotide sequences from 7 housekeeping genes (bootstrap 1000 replicates). Two non-Bcc *Burkholderia* species and a *Ralstonia pickettii* strain were used as outgroups. The distances for nucleotide data were inferred using the General Time Reversible (GTR) model with gamma distribution (5 rate categories and 49% invariable sites). Scale bar indicates 0.1 substitutions per site. The presence or absence of *shvR* and the *afc* gene cluster has been indicated with + or -. *B arboris* strain has not yet been fully sequenced and the presence of *shvR* or *afcE* could not be determined. The strains of each species are clustered except for two *B*. *cepacia* strains (GG4 and JBK9). *B*. *stabilis* LA20W and *B*. *vietnamiensis* strain AU4i. We propose that their classification may need to be reviewed given the following reasons. The strain *B*. *cepacia* GG4 clusters closer to *B*. *ambifaria* species as has already been reported [61]; the clustering of *B*. *cepacia* strain JBK9 together with *B*. *stabilis* is consistent with the 73% whole genome similarity shared by the *B*. *cepacia* JBK9 and *B*. *stabilis* strains [52]. The *B*. *stabilis* strain LA20W not only has higher genome similarity with *B*. *pyrrocinia* DSM 10685 (91.25%; while only 67.2% with the *B*. *stabilis* reference strain ATCC BAA-067 (or LMG14294) [52]), but also encodes the *shvR*/*afc* cluster sequence in its genome (S3 Fig), while *B*. *stabilis* ATCC BAA-67 does not. *B*. *vietnamiensis* AU4i is the only one that appears clustered with *B*. *cepacia* ATCC 25416, being consistent with high similarity between their genomes 83–86% (against 52–54% similarity between AU4i strain and other *B*. *vietnamiensis* strains) [52] and the presence the *shvR*/*afc* cluster in its genome (S3 Fig).

To better understand a role for ShvR/Afc in virulence within the Bcc, we performed bio-informatics analysis. The *Burkholderia cepacia* complex has seen a rapid increase in the number of bacterial species it contains due to improved characterization and reclassification [32]. The current methods for discrimination between species are based on *recA* gene sequences, multilocus sequencing and whole genome studies (reviewed in [33]). At present, the complex includes 21 formally named species [1,2]. For the construction of a phylogenetic tree, representative genomes of each species were included (in some cases more than one strain) with either a complete genome and annotation status or whole genome sequence (WGS, see S2 Table). A phylogenetic inference tree of these species was constructed using the nucleotide sequences of the Multilocus Sequence Typing (MLST) housekeeping genes (*atpD*, *gyrB*, *gltB*, *lepA*, *recA*, *phaC* and *trpD*) [34]. Fig 8 shows a maximum-likelihood phylogeny generated from concatenation of these 7 genes. *B*. *pseudomallei* K96243, *B*. *thailandensis* E264 and *Ralstonia pickettii* 12J were used as out-groups.

To better understand the evolution of the LTTR regulator ShvR within the complex, BLAST-analysis was performed to identify orthologs of the gene BCAS0225 from *B*. *cenocepacia* J2315. A gene with high similarity to BCAS0225 (identity >85%) was identified in a subclade containing strains from 10 out of the 20 species for which complete genome sequences are available (*B*. *arboris* has not been sequenced). In all cases, the gene was located on the megaplasmid pC3. Similarly, orthologs were identified for *afcE* (BCAS0208). Fig 8 shows that the presence/absence of *shvR* within the Bcc complex matches that of *afcE*. Further investigation of the adjacent *afc* cluster showed that the 24 *afc* genes and their organization, including the presence and location of the *shvR* gene are well-conserved among *shvR*-containing Bcc species (S3 Fig).

In addition to the 19 analysed Bcc strains (covering 10 species) that carry the *shvR*-*afc* cluster, *B*. *pseudomallei* and *B*. *mallei* have a subset of the *afc* genes, including *afcE*. The operon in *B*. *pseudomallei*, located on chromosome 1, shares similarity to 15 of the 22 genes in the Bcc *afc* cluster and is likely regulated by an LTTR regulator unrelated to ShvR, encoded by an ORF adjacent to the cluster (BPSL0494, S3 Fig). This regulator shares amino acid similarities with only the DNA binding domain of the ShvR protein (27% amino acid identity). BPSL0494 shares 77% nucleotide identity with BCAS0283, another LTTR identified in *B*. *cenocepacia* J2315 encoded on pC3, with homology mainly in the 5’ 200 bp and the 3’ 150 bp. A maximum-likelihood phylogeny of *afcE* (S4 Fig) confirmed that the acyl-CoA dehydrogenase from *B*. *pseudomallei* is the most distant in the *Burkholderia* group, although they share 80% of nucleotide similarity.

Analysis of the region upstream of the *shvR* coding sequence amongst the *shvR*-positive strains of the Bcc complex showed nucleotide variations (SNPs, indels), not only between species but also between strains belonging to the same species (S5 Fig). As expected, strains belonging to the same species contain fewer variations, compared to, for example, those observed between the *B*. *cenocepacia* and *B*. *lata* strains. Within the *B*. *cenocepacia* species, the *recA* lineage IIIA and IIIB (Fig 8) each appear to have their own consensus sequence for the *shvR* upstream region. Interestingly, a 2-nucleotide gap, at position -238 from the translation start site (S5 Fig), followed by a short highly non-homologous region compared to the conserved sequence found in the other cluster, is observed in the strains of *B*. *cepacia*, *B*. *pyrrocinia*, *B*. *stabilis*, *B*. *lata*, *B*. *contaminans*, *B*. *metallica* and *B*. *vietnamiensis*. This is in agreement with the phylogenetic relationship shown in Fig 8, and suggests that this deletion and the following non-homologous region occurred before speciation.

### Overexpression of *shvR* in H111 causes an *afc*-dependent increase in virulence

**Fig 9:**
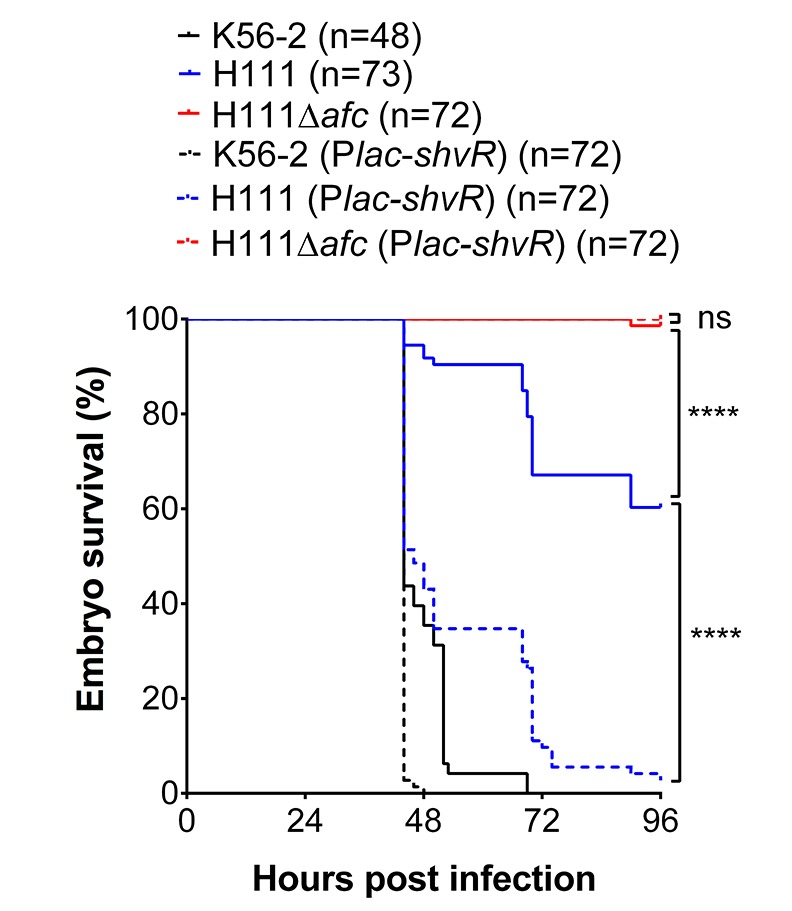
Differences in *shvR* expression between *B*. *cenocepacia* strains K56-2 and H111 cause *afc*-dependent differences in virulence. Embryo survival after intravenous injection of an average of 192 ± 22 CFU of *B*. *cenocepacia* K56-2 wildtype (solid black lines), 273 ± 132 CFU of K56-2(P*lac*-*shvR*_pBBR_) (dashed black lines), 185 ± 47 CFU of *B*. *cenocepacia* H111 wildtype (solid blue lines), 174 ± 10 CFU of H111(P*lac*-*shvR*_pBBR_) (dashed blue lines), 198 ± 53 CFU of H111Δ*afc* (solid red lines) and 228 ± 29 CFU of H111Δ*afc* (P*lac*-*shvR*_pBBR_) (dashed red lines). Pooled results from three independent experiments are shown (n = 24 per experiment per strain, K56-2 was included only in 2 experiments). Statistical analysis was performed using a Log rank (Mantel-Cox) test. **** p < 0.0001, ns: not significant.

*B*. *cenocepacia*, *B*. *cepacia* and *B*. *contaminans*, Bcc species which are generally correlated with more severe disease in humans, all carry genes encoding ShvR and Afc proteins. Several *B*. *cenocepacia* strains, including the epidemic CF isolates K56-2 and J2315, are highly virulent in different model systems, including zebrafish [29]. However, *B*. *cenocepacia* H111, which is very closely related to K56-2, was shown to be less virulent in *G*. *mellonella* and rats [35,36]. In zebrafish, we previously described that this strain can show substantial variation between experiments [37], with mortality rates between 20 and 60% at 4 days post fertilisation (dpf), but always less virulent than K56-2 (Fig 9). We also found that H111 does not show *afc*-dependent antifungal activity as seen for K56-2 [37]. Comparison of the *shvR* coding sequences showed 3 silent SNPs between H111 and our K56-2 strain. Of note, the sequenced K56-2Valvano strain has a 4^th^ SNP giving rise to a variant Tyr78 to Cys78. We set out to analyse whether differences in *shvR* expression could account for the differences in virulence observed between K56-2 and H111, and thus confirm the role of *shvR*/*afc* in virulence of *B*. *cenocepacia*.

To be able to study the role of *shvR* and *afc* in virulence of H111 in the absence of upstream regulatory signals, we cloned the *shvR* gene of H111 under control of the *lac*-promoter on pBBR1MCS and constructed an H111 mutant lacking the complete *afc* cluster. K56-2, H111 and H111Δ*afc* were transformed with P*lac*-*shvR*_pBBR_ or the pBBR1MCS control plasmid. Expression of *shvR* from the *lac* promoter led to a change in colony morphotype of H111 from shiny to rough, in a manner that depended on *afc* (S6 Fig). This suggests that the endogenous *shvR* gene is not expressed in H111 on agar plates, but that expression of *shvR in trans* induces the expression of a functional AFC lipoprotein. H111 caused fatal disease in on average 40% of injected embryos over a 4-day time period, compared to 100% mortality in 2 to 3 days for K56-2 (Fig 9). Zebrafish embryos injected with the H111Δ*afc* mutant survived and remained without any clinical signs of infection during the experimental time, in agreement with the experiments performed with K56-2Δ*afcE* (Fig 6B). The finding that *afc* is essential for acute fatal infection also in H111 suggests that *shvR* and *afc* expression is induced in wildtype H111 upon injection in zebrafish larvae, but probably does not reach the level of that in K56-2. Strikingly, overexpression of *shvR* from the *lac* promoter increased virulence in H111, but not the H111Δ*afc* mutant, almost to that seen with *B*. *cenocepacia* K56-2 (Fig 9). These data validate the results obtained with K56-2 that *shvR*/*afc* are critically important for acute virulence in zebrafish larvae. They also unambiguously demonstrate that *shvR*/*afc* are functional in H111, and suggest that differences in inducing signals or upstream elements render H111 less virulent in zebrafish compared to K56-2.

## Discussion

The LysR-type transcriptional regulator ShvR has been shown to tightly control the expression of an adjacent operon, called *afc*, involved in antifungal activity [25]. The zebrafish embryo model, in which persistent and acute infection caused by different Bcc strains can be studied in detail in the context of an innate immune response [29,30] was exploited to gain better insight into a role for ShvR and AfcE in virulence of *B*. *cenocepacia* K56-2. We found that while *shvR* and *afcE* are not required for bacterial persistence in the host, they are both essential for the induction of a robust fatal pro-inflammatory infection. Our study shows that ShvR and AfcE are key elements in the transition from an intracellular bacterial stage to disseminated pro-inflammatory infection in zebrafish larvae.

A previous microarray study has shown that the expression of the *afc* cluster is under tight positive regulatory control of ShvR [25]. The *afc* cluster consists of 24 genes arranged in two divergently expressed operons that specify the synthesis of a lipopeptide located in the membrane of the bacterial cells, previously identified as AFC BC11 [26]. The role of several genes in the *afc* cluster has been analysed in more detail. While *afcA*, *afcB*, *afcC*, *afcD*, *afcE* and *afcF* have been shown to be important for antifungal activity against *Fusarium solani*, BCAS0204 (ABC transporter) and BCAS0207 (citrate synthase) are dispensable [26,27]. In addition, AfcE and AfcF, the latter being a putative FAD-dependent oxidoreductase, but not BCAS0204 and BCAS0207 were involved in virulence in alfalfa seedlings. Previous studies have further shown that Δ*afcE* and Δ*afcF* mutants have altered membrane lipid profiles, although additional membrane properties, including membrane permeability and membrane morphology, were affected only in the Δ*afcE* mutant [27]. Additionally, *B*. *cenocepacia* lacking *afcE* produces less biofilms and displays a shiny colony morphology [27], similar to the phenotypes observed for Δ*shvR*. The similarity in observed phenotypes between the Δ*shvR* and Δ*afcE* mutants suggests that the *afc* operon is an important regulatory target of ShvR, with a significant role for the *afcE* gene product.

Recently, we showed the essential role of macrophages in the establishment of a replicative niche for *B*. *cenocepacia* K56-2 and in the development of a robust pro-inflammatory response that becomes rapidly fatal for zebrafish larvae [29]. In contrast, infection with the Δ*shvR* and Δ*afcE* mutants was not fatal and led to only minimal induction of inflammatory responses (Figs 3 and 6). Importantly, after phagocytosis by macrophages, the Δ*shvR* and Δ*afcE* mutants did not disseminate and total bacterial burden only slightly increased over time, in contrast to the dissemination and rapid increase in bacterial numbers seen for the wildtype [30]. However, the absence of *shvR* and *afcE* did not prevent the bacteria from replicating intracellularly (Fig 2 and S2 Fig). These data show that *shvR* and *afcE*, although not required for persistence and replication in macrophages, are critical for the development of fatal pro-inflammatory disease. This is in agreement with results obtained in a chronic lung infection model in rats, where Δ*shvR* and Δ*afcE* mutants showed significant reduction in lung inflammation, while bacteria persisted in the lungs, sometimes replicating to higher numbers than the wildtype [24,28]. We are now investigating in more detail the virulence profile of mutants in other genes of the *afc* operon, and whether general changes in membrane properties or a dysfunctional AFC lipopeptide itself, caused by the absence of AfcE, are responsible for the observed lack in mounting a robust pro-inflammatory response by *B*. *cenocepacia* K56-2.

To test our hypothesis that ShvR and AfcE have an important role in the transition from intracellular persistence to acute infection we used a rhamnose-inducible expression system [31]. Our data show that the temporal induction of *shvR* and *afcE* expression in intracellular Δ*shvR* and Δ*afcE* bacteria, respectively, restored properties that are essential for bacterial dissemination and induction of pro-inflammatory responses. Rhamnose-mediated activation of *shvR* and *afcE* resulted in neutrophil recruitment and increased global expression of *cxcl8* and *il1b* in the embryos (Figs 5 and 7). The infection rapidly worsened and became fatal for the zebrafish embryos (Figs 4B and 7A), confirming our hypothesis that ShvR and AfcE are essential for persistent to acute transition in this model.

Bacteria belonging to the *Burkholderia cepacia* complex (Bcc) are life threatening opportunistic pathogens of cystic fibrosis patients and immunocompromised people. One of the major concerns in CF infections is the recurrent acute exacerbations during infection that severely deteriorate the health of the patients with often fatal consequences. The underlying signals and mechanisms that regulate pathogenesis and lead to chronic-acute transitions are not known. Studies on transcriptional regulators of *Pseudomonas aeruginosa*, including AmpR and RetS/LadS/GacS, have shown that these are involved in regulating the switch between acute to chronic infection [19,38–42]. Our study describes for the first time a regulator that is absolutely required for *B*. *cenocepacia* to change a persistent intracellular lifestyle to acute pro-inflammatory infection. The data strongly suggest that the downstream regulated target of ShvR that is responsible for the acute infection is the *afc* gene cluster, with a major role for *afcE*.

Our bioinformatics analysis shows that *shvR* and the *afc* cluster are present in the genomes of 10 of the 20 Bcc species of which the whole genome sequence has been published (Fig 8). It is interesting that the *shvR*/*afc* cluster is present in most of the more virulent species in the complex including *B*. *cenocepacia*, *B*. *cepacia* and *B*. *contaminans*, while *B*. *stabilis* LMG14294 for example, which causes persistent infection in the zebrafish model [30] and is reduced virulent in a rat model [43], lacks *shvR*/*afc*. In contrast to differences in the flanking gene sequences between strains, *shvR* and *afc* have evolved as one unit with a highly conserved gene structure over time (S3 Fig), suggesting an important evolutionary advantage of ShvR-dependent production of the AFC lipopeptide. Interestingly, the important human pathogens *B*. *pseudomallei*, the causative agent of melioidosis or Whitmore disease, and *B*. *mallei*, causing glanders, encode a subset of the *afc* genes. Although an *afcE* homolog is present, the LTTR encoded adjacent to the *afc* cluster is not an ortholog of *shvR*. The presence of the *afc* cluster also in the *B*. *pseudomallei* cluster suggests an ancestral origin that has adapted through divergent evolution in the *B*. *pseudomallei* and Bcc clusters, with loss of the cluster several times during speciation of the Bcc. Although less likely, the *shvR*/*afc* cluster may have been acquired independently during evolution in the Bcc and the *B*. *pseudomallei* cluster. It would be interesting to know whether *afcE* has a similar role in virulence in *B*. *pseudomallei* and *B*. *mallei*.

O’Grady and colleagues have demonstrated that *shvR* is continuously expressed during growth of *B*. *cenocepacia* K56-2 in LB, with a peak in expression between 8 and 18 h [25], however the signals that may either inhibit or induce its expression, including those during infection of humans and in the natural environment, are not known. Acidic pH, low aeration and/or growth on a surface to stationary phase have been suggested to be important conditions for maximal AFC production in *B*. *pyrrocinia* BC11 [26]. *B*. *cenocepacia* K56-2 of the ET12-lineage is one of the most virulent Bcc strains in different animal models and the constitutive and high expression profile of *shvR* in this strain under different conditions may represent a key factor for the acute and disseminated character of infection by this and other isolates of the epidemic ET12 lineage.

Interestingly, for *B*. *cenocepacia* H111 it was recently shown that the *afc* cluster was not involved in the antifungal activity observed from this strain, due to lack of expression [37,44]. In addition, although H111 is more virulent than K56-2 in the *C*. *elegans* infection model since it has the nematocidal protein AidA [45], virulence of H111 in *G*. *mellonella* [37] and zebrafish (Fig 9) is reduced compared to that of K56-2. Nonetheless, H111 is more virulent than strains including *B*. *stabilis* and *B*. *vietnamiensis*, which cause persistent infection in zebrafish [29], and can still cause fatal infection in a manner that is totally dependent on *afc*. This suggests that *shvR*-inducing signals must be present during infection in zebrafish and *G*. *mellonella*, but that expression levels are sub-optimal compared to K56-2, since overexpression of *shvR* from the *lac* promoter increases virulence to H111 to almost K56-2 levels. Our results with H111 validate that regulation and expression of ShvR play a major role in the *afc*-dependent acute virulence of *B*. *cenocepacia*. These results are also consistent with the observations that the CF patient from which H111 was isolated did not show acute symptoms and the infection was cleared after 6 months without changing the therapy regime [46]. Overall, differences in *shvR*/*afc* gene regulation, or perception and type of environmental cues, may contribute to differences in antifungal activity and/or virulence. A better understanding of these factors may reveal how Bcc bacteria rapidly adapt to different environmental conditions and may provide new insights into the virulence potential of *B*. *cenocepacia* strains in humans.

This study emphasizes that properties which are important for the bacteria to thrive in the environment, may be important virulence factors in opportunistic infections. The role of lipids and lipoproteins in virulence, for instance through adherence to host cells and modulation of inflammatory processes has been recognised for many years, making for potential vaccines. New treatment strategies may be designed by improving our understanding of how *Burkholderia cenocepacia* resists clearance from macrophages and adapts for persistence, or, is able to cause acute disease in interaction with macrophages. We are currently studying the cellular mechanism of AFC-dependent induction of pro-inflammatory responses, and propose ShvR/AFC as a novel target to reduce pro-inflammatory responses during Bcc infection.

## Materials and methods

### Ethics statement

Zebrafish (*Danio rerio*) were kept and handled in compliance with the guidelines of the European Union for handling laboratory animals (http://ec.europa.eu/environment/chemicals/lab_animals/home_en.htm). Zebrafish studies performed at VBMI are approved by the Direction Départementale de la Protection des Populations (DDPP) du Gard (ID 30-189-4) and the Comité d'Ethique pour l'Expérimentation Animale Languedoc-Roussillon (CEEA-LR-12186). Infection experiments in this study were terminated before the larvae reached the free feeding stage and did not classify as animal experiments according to the 2010/63/EU Directive.

### Zebrafish

The zebrafish line AB was used as wildtype (WT), and the transgenic reporter lines Tg(*mpx*:*eGFP*)^*i114*^ [47] and Tg(*mpeg1*:*mCherry-F*)^ump2Tg^ [48] were used to analyse host phagocyte behaviour. Care and maintenance of zebrafish was as described previously [29]. Eggs were obtained by natural spawning and incubated at 29°C in Petri dishes containing E3 medium (5mM NaCl, 0.17mM KCl, 0.33mM CaCl_2_, 0.33 mM MgSO_4_) as described [49]. Methylene blue was omitted.

### Bacterial strains, plasmids and growth conditions

The bacterial strains, plasmids and primers used in this study are listed in S1 Table. Strains were grown at 37°C in Lysogeny Broth (LB), supplemented with ampicillin at 100 μg/mL for *Escherichia coli*, chloramphenicol at 30 μg/mL and 100 μg/mL for *E*. *coli and Burkholderia cenocepacia* respectively, tetracycline at 250 μg/mL (*B*. *cenocepacia*) and trimethoprim at 50 μg/mL (*B*. *cenocepacia*). No differences in growth were observed between *B*. *cenocepacia* K56-2 and the *ΔshvR* and *ΔafcE* mutants when grown in LB medium.

### Generation of plasmid constructs

For the construction of pIN233, the *mCherry* coding region from plasmid pSAT1:mCherry-MCS-nVenus (pE3370) was amplified using primers mCherry-3 and mCherry-4. The PCR fragment was digested with *Xba*I and *Nde*I and cloned into pIN29 [30], replacing the *DSRed* coding sequence. Plasmid pIN298 resulted from cloning the *mCherry* coding sequence from pIN233 into pCR11 (a gift from M. Kovach), a Cm^R^ derivative of the single copy plasmid pMR10 (GenBank: AJ606312.1), using *Hind*III and *Xba*I restriction sites.

A ~1.6 kb fragment containing full length *shvR* and upstream region P*shvR* (primers pshvRXhoI for and shvRXbaI rev), a 995 bp fragment containing the *shvR* coding region (primers shvRNdeI for and shvRXbaI rev) and a ~1.8 kb fragment containing the *afcE* coding region (primers 0208_NdeI for and 0208_XbaI rev) were separately amplified from K56-2 genomic DNA by PCR using *Pfu* polymerase (Life technologies, USA). The fragments were first subcloned in pUC29 and verified by sequencing (MWG Operon Eurofins, Germany).

To construct pIN308, the PCR product of P*shvR*:*shvR* was cloned into pIN29 as a *Xho*I/*Hind*III fragment, and named pIN307. From this plasmid, the P*shvR*:*shvR* fragment was cloned into pIN298 using the *Spe*I and *Bam*HI restriction enzymes (*Xho*I and *Xba*I).

The rhamnose inducible system from pSCPrhaB2 [31], containing the rhamnose-regulated P*rhaB* promoter and the genes *rhaR* and *rhaS* of the rhamnose operon, was cloned into pIN177 using *Xba*I and *Pst*I restriction sites, and named pIN299 (or P*rhaB-*, serving as negative control in rhamnose induction experiments, for simplicity). pIN177 is a derivative of pBBR1MCS plasmid lacking the *oriT*/*mob* region and containing a strong *trp* termination signal (pIN32; [30]), a *tac* promoter sequence (*Pst*I/*Nde*I fragment from pIN17; [30]) and an additional *Nde*I/*Xba*I linker (catatgaagctttcgcgagctcgagatctaga).

To create P*rhaB*:*shvR* (pIN310), allowing rhamnose-inducible expression of *shvR*, the *shvR* coding sequence was cloned downstream the P*rhaB* promoter sequence as follows: the 995 bp *shvR* PCR fragment (primers shvRNdeI for and shvRXbaI rev) was cloned into *Xba*I/*Nde*I-digested pIN299, and named pIN309. A P*tac*:mCherry fragment from pIN233, cloned in pUC29 as a SpeI/NotI fragment, was then inserted in the *Kpn*I site of pIN309, resulting in pIN310. Plasmid pIN311 (P*rhaB*:*afcE*) was constructed by digesting pIN299 with *Nde*I/*Xba*I restriction sites and inserting the *afcE* fragment amplified using the primers 0208_NdeI for and 0208_XbaI rev.

The *afcE* complementation plasmid pINR139 was generated by cloning a P*tac*:DSRed fragment from pIN29 as a *Spe*I/*Sac*I fragment into pBBRS0208.

Plasmids were transferred into *B*. *cenocepacia* K56-2, Δ*shvR and* Δ*afcE* mutants by electroporation as described earlier [30].

The plasmid P*lac*-*shvR*_pBBR_ carries the coding sequence of *shvR* amplified from *B*. *cenocepacia* H111, using primers shvRHindFor and shvRBamRev. The restriction sites *Bam*HI and *Hind*III were used to clone the fragment in pBBR1MCS.

### Deletion of the *afc* cluster from *B*. *cenocepacia* H111

Deletion of the *afc* cluster was carried out using vectors pSHAFT2-FRT and pEX18Tp-FRT, as described in [37]. A fragment adjacent to the region to be deleted (UP) was amplified from *B*. *cenocepacia* H111 with primers upXhoF and upBglIIR (~1.3 kb), and digested with *Xho*I and *Bgl*II. The fragment was then inserted in pSHAFT2-FRT using the same sites, resulting in pSHAFT2-FRT-afcUP. This plasmid was inserted into the genome of *B*. *cenocepacia* H111 by single crossover recombination. Correct integration was confirmed with primer pairs AFCdelUPcheckF (GATCATCTTCTTCTCGCTCG) and pSHAFTseqR4 (GAACACTTAACGGCTGACAT, ~1.5 kb) and pSHAFT2For3 (GATTATTTTGCCCCGGTTTT) and AFCdelUPcheckR (GGAGATTTCGCATGATGTTT, 2 kb).

A fragment bordering the *afc* cluster on the other side (DOWN) was amplified from *B*. *cenocepacia* H111 using primers downpstF and downBamR. The resultant ~1.3 kb fragment was digested with *Pst*I and *Bam*HI and inserted between these sites in pEX18Tp-FRT. This vector (pEX18Tp-FRT-afcDOWN) was inserted into the genome of *B*. *cenocepacia* H111 bearing the previously integrated pSHAFT2-FRT derivative by single crossover recombination. Correct integration was confirmed with primer pairs AFCdelDOWNcheckF (GAATTGAACCGCTATCGCC) and M13R (~1.4 kb) and M13F and AFCdelDOWNcheckR (GCACAGGTTGCAGGTATT, 1.7 kb).

Plasmid pBBR5::FLP [50] was then introduced by conjugation, to stimulate recombination between the FRT sites integrated into the genome.

Flippase-mediated recombination was carried out at 30°C, and colonies were selected using the marker present on the pBBR5::FLP derivative. Colonies were then checked for loss of the markers present on the pSHAFT-2FRT and pEX18Tp-FRT derivatives (chloramphenicol and trimethoprim, respectively). Finally, deletion was confirmed by amplifying across the ends of the deleted region with primers AFCDELcheckFor and AFCDELcheckRev. Successful deletants gave rise to a band of ~700 bp.

### Microinjection conditions and rhamnose-mediated induction of gene expression

Microinjection of zebrafish embryos was performed as previously described [49]. Briefly, *B*. *cenocepacia* strains were grown overnight in LB broth with appropriate antibiotics at 37°C. Bacterial dilutions to obtain the desired inoculum concentration were prepared in PBS (with 0.05% phenol red to visualize microinjection). Embryos were dechorionated 2 hours prior to microinjection and at 30 hours post fertilization (hpf) they were injected in the blood island. For microinjection, embryos were placed on agarose plates containing E3 medium with 0.02% buffered MS222 (tricaine; ethyl-3-aminobenzoate methanesulfonate salt). Injection was performed with a Femtojet microinjector (Eppendorf) and a micromanipulator with pulled microcapillary pipettes, under a stereo light microscope (Leica MS5). The pool of infected embryos was then randomized over CFU plating and survival assays. Embryos were maintained individually in 48-well plates in E3 medium at 29°C. In order to specifically induce the expression of *shvR* and *afcE* in Δ*shvR* and Δ*afcE* mutants, respectively, at 24 h after injection of the bacterial inoculum, rhamnose (2% in PBS) was injected directly in the blood circulation. As a negative control, half of the embryos of each group were injected with PBS. The embryos were then kept in E3+2% rhamnose or in E3 medium, respectively. Immediately after bacterial injection (T = 0 hpi), 5 embryos per strain were disrupted and plated on LB agar with the appropriate antibiotics to determine the precise inoculum size. At 24, 48 and 72 hpi (in the rhamnose assays), 10 μl of each serial dilution was deposited on a squared LB-agar plate for determination of CFU. Embryo survival was determined at regular time points, starting at 42 or 44 hpi and every two hours during periods with high mortality rates. Time of death was based on the absence of a heartbeat.

### RNA extraction, cDNA synthesis and quantitative RT-PCR

RNA extraction, cDNA synthesis and qPCR analysis of zebrafish genes were performed as described [49]. The peptidylprolyl isomerase A-like (*ppial*) gene was used as a reference gene. The ΔΔCt method was used for analysis of the data, represented as column bar graphs normalized to a PBS-injected control group at each time point. Three biological replicates were performed (unless mentioned otherwise), each with two technical replicates. So far we have been unable to optimise qPCR of bacterial genes from infected zebrafish larvae, due to the high ratio of host to bacterial RNA.

### Microscopy, image processing and quantification of fluorescent host immune cells

Embryos were imaged using a Leica DM IRB inverted microscope coupled with a Coolsnap fx black and white camera (Roper Scientific) as described [49], or a Nikon AZ100, coupled with Coolsnap HQ2 (Roper Scientifique). MetaVue software was used for imaging. Adobe Photoshop was used to colour black and white images, prepare overlay images taken with different channels, include scale bars, and crop images for the purpose of showing enlargements/insets. For analysis of phagocytosis and quantification of intracellular Δ*shvR* bacteria (sample image Fig 2A), the embryos were fixed in 4% PFA for 2 hours at RT, or overnight in 3% PFA at 4°C and analysed using an Olympus confocal laser scanning microscope Fv10i and Fluoview software.

Quantification of neutrophil cell numbers was performed according to [51]. In brief, images taken with identical camera settings (S1A Fig) were converted to binary in ImageJ 1.47v, resulting in images in which fluorescence was converted into black pixels onto a white background (S1 Fig). Five randomly selected individual cells per image (embryo) were taken to determine pixel size per phagocyte, as described [51]. Total pixel counts were then divided by the average of the 5 individual cells to determine the total number of fluorescent cells.

### Phylogenetic analyses of *Burkholderia* species

A maximum likelihood phylogeny was created from 43 *Burkholderia cepacia* complex strains and 3 outgroup species (*B*. *pseudomallei* K96243, *B*. *thalandensis* E264 and *Ralstonia pickettii* 12J, in S2 Table). The tree is based on the concatenated alignment of fragments of nucleotide sequences of the Multilocus Sequence Typing (MLST) gene set, *atpD*, *gyrB*, *gltB*, *lepA*, *phaC*, *recA* and *trpB* [34]. For strains with MLST ID indicated in S2 Table, the sequences were retrieved from PubMLST database (pubmlst.org/bcc), for the others the coding sequence of the genes were obtained from BLAST searches in NCBI [52], PATRIC (patricbrc.org, [53]) or the *Burkholderia* Genome Database (burkholderia.com, [54]). For those cases, the sequences were shortened upon the alignment. The sequences of each gene were first aligned in MEGA6.06 [55] using ClustalW [56,57] and then all gene sequences were concatenated using the program SequenceMatrix [58]. The concatenated genes sequences were then introduced in MEGA6.06 to construct a maximum likelihood tree. The General Time Reversible (GTR) model [59] was used to determine nucleotide distances, with gamma distribution (5 rate categories and 49% invariable sites), assuming partial deletions in the missing data and a cut-off of 95%. The maximum likelihood heuristic method used was the Nearest-Neighbour-Interchange (NNI). A bootstrap of 1000 replicates was used.

### Phylogenetic analysis of *shvR* and *afcE*

A BLAST analysis of the promoter region of *shvR* (500bp) from *B*. *cenocepacia* J2315 (BCAS0225) was performed using PATRIC against a genome database with the *Burkholderia* strains summarised in S2 Table (except for *B*. *arboris* LMG 14939). The sequences of each of the identified genes were obtained (either from PATRIC, NCBI or *Burkholderia* Genome Database) and aligned in MEGA6.06 [55] using ClustalW [56,57]. A maximum likelihood phylogenetic tree for the *afcE* gene was created in MEGA6.06 using the same parameters as described above for the *Burkholderia* species tree, except that the model used was Tamura 3-parameters model (T92), with gamma distribution (5 rate categories), and were considered 40% invariable sites.

### Analysis of the *Afc* cluster

To analyse the similarities of the *afc* clusters of *Burkholderia* species, the pC3 genomic sequences (or contigs) of the 43 sequenced Bcc strains (except for *B*. *arboris* LMG 14939) and *B*. *pseudomallei* K96243, summarized in S2 Table, were retrieved from NCBI. Sequence alignments were performed with MAUVE using default parameters (default seed weight set to 15; set for determination of Local Collinear Blocks (LCB); full alignment (default minimum LCB weight); set for iterative refinement; set for sum-of-pairs LCB scoring) [60].

### Statistical analyses

For statistical analysis GraphPad Prism 6.0 software was used. The average inoculum is calculated as the average CFU of 5 embryos T = 0 values ± SD, and indicated in the legend to the respective graphs. CFU counts from individual larvae at later time points were log_10_ transformed and the significance between the multiple selected groups was determined using one-way ANOVA with Sidak’s Multiple Comparisons test. To include larvae in which no CFU were detected at 24 or 48 hpi, the 0 count was converted to 1, prior to log-transformation. Since the geometric mean cannot be calculated for groups that contain log_10_ values of 0, we have set this value at 0.0001 to be able to calculate the geometric mean. No difference in significance was found when using the 0 or 0.0001 log value.

In survival assays statistical analysis was done using a Log rank (Mantel-Cox) test. For statistical analysis of macrophage and neutrophil cell counts and intracellular bacterial numbers, one-way ANOVA with Sidak’s Multiple Comparisons test, unpaired t-test, and Mann-Whitney were used as indicated in the legends to each graph.

The data from qRT-PCR of cytokine genes were log_2_-transformed, and significance of the data was analysed using one-way ANOVA with Tukey’s Multiple Comparison Test. Columns indicate mean fold with SEM. For each treatment, normalized to the corresponding PBS control, significance in relative fold-change is indicated with an asterisk above the column, significance between treatments is indicated with a connective line between the bars. In rhamnose assays, rhamnose-induction and PBS treatments in both conditions (control plasmid and gene expressing plasmid) were normalized to the non-infected PBS control. Significance is indicated as: ns, non-significant, *, p ≤ 0.05; **, p ≤ 0.01; ***, p ≤ 0.001; ****, p ≤ 0.0001.

## Supporting information

S1 Fig Related to Fig 3B. Quantification of fluorescent host immune cells during infection. **(A)** Representative images at 0, 24, 43 and 72 hpi of Tg(*mpx*:e*GFP*) embryos, non-infected and infected with *B*. *cenocepacia* K56-2, Δ*shvR*, and the complemented Δ*shvR* mutant. Scale bars, 50 μm. **(B)** Representative images of binary conversion of fluorescent microscopy images of Tg(*mpx*:e*GFP*) embryos, non-infected and infected with K56-2. Scale bars, 50 μm.
(TIF)

S2 Fig Related to Figs 2A, 2B and 6. Quantification of intracellular Δ*afcE* bacteria. **(A)** Quantification of intracellular Δ*afcE* bacteria in PFA-fixed embryos at 1 hpi over the yolk as indicated in Fig 2A for Δ*shvR* using confocal microscopy. Each data point represents an individual Tg(*mpeg1*:*mCherry-F*) transgenic embryo injected intravenously with wildtype *B*. *cenocepacia* K56-2 (black dots, n = 13) or Δ*afcE* (red dots, n = 14) expressing eGFP. Data represent the same experiment as shown in Fig 2B, with the same values for K56-2. Counts per embryo are presented as percentage of internalized bacteria relative to total numbers of bacteria in the yolk sac region. A total of 220 and 122 bacteria were counted for K56-2 and Δ*afcE*, respectively, in 15 embryos. Unpaired t-test, with mean 86.7 ± 5.3 and 84.7 ± 7.3, respectively, p-value 0.84. Data are representative of two independent experiments. **(B)** Quantification of the number of intracellular Δ*afcE* bacteria per macrophage in PFA-fixed embryos at 1 and 24 hpi, presented as frequency distribution histogram. Data represent the same experiment as shown in Fig 2E. The graph represents a total of 197 (14 embryos) and 68 macrophages (12 embryos), at 1 hpi (mean: 1.4 ± 0.07 bacteria per macrophage) and 24 hpi (10.1 ± 1.3), respectively. Mann Whitney test p < 0.0001. Representative of 2 experiments.
(TIF)

S3 Fig Related to Fig 8. Comparison of the *afc* cluster across Bcc strains encoding *shvR*. Bioinformatics analysis of the *afc* cluster and flanks reveals a similar organization in all the 10 species encoding *shvR*, with variations in the flanking genes. In *B*. *pseudomallei* chromosome 1 a similarly structured cluster is present, although it is shorter and regulated by a LTTR that differs from *shvR* (dark green arrow). Homology between the *afc* cluster and the genes in *B*. *pseudomallei* is indicated with grey shades. *shvR* is indicated in green, *afcE* in orange and the other genes of the *afc* cluster in blue. Blue empty arrow indicates a putative coding sequence that has not been annotated in all strains (BCAS0200). Black arrows indicate the flanking genes of the *shvR*-*afc* cluster: the C4-dicarboxylate ABC transporter operon (BCAS197-BCAS199) on the left, and a hydrolase-encoding gene (BCAS0226) and an MFS transporter (BCAS0227) on the right side. Grey arrows represent a 6-phosphogluconolactanase encoding gene and a chemotaxis protein is marked with a light blue arrow. Schematic representation of the genes is not to scale.
(TIF)

S4 Fig Related to Fig 8. The Bcc *afcE* gene is also encoded by *B*. *pseudomallei* species. Maximum likelihood phylogeny tree was inferred based on the alignment of *afcE* nucleotide sequences in Bcc and *B*. *pseudomallei* (bootstrap 1000 replicates). The distances for nucleotide data were inferred using the Tamura 3-parameter model, with gamma distribution (5 rate categories) and 40% invariable sites. Scale bar indicates 0.2 substitutions per site.
(TIF)

S5 Fig Related to Fig 8. Alignment of the upstream non-coding region of *shvR* in *shvR*-encoding Bcc species. The sequences shown are only 268 bp of the 617 bp intergenic region between *afcD* and *shvR*. The nucleotide sequence located upstream of *shvR* has several SNPs in the other 18 *shvR* encoding Bcc strains compared to *B*. *cenocepacia* J2315 (see Fig 8). Between the different *B*. *cenocepacia* strains the similarity ranges from 87 to 99% identity (the weakest similarities are observed for the strains HI2424, MC0-3 and AU1054). Compared with the other Bcc species, the similarities range from 76% to 81%. The 2-nucleotide gap at -238 bp from the translation start site (ATG) is indicated with black vertical arrows. The black bar indicates a short highly non-homologous region compared to the conserved sequence found in the *B*. *cenocepacia* J2315.
(TIF)

S6 Fig Related to Fig 9. Overexpression of *shvR* changes H111 colony morphology from rough to shiny in an *afc*-dependent manner. Effect of expression of P*lac-shvR* on morphology of individual colonies, visualised using an AZ100 microscope.
(TIF)

S1 Table Related to experimental procedures. Bacterial strains, plasmids and primers used in this study.
(DOCX)

S2 Table Related to Fig 8. *Burkholderia cepacia* complex strains and outgroup species used in this study. For each strain the genome status (WGS stands for Whole Genome Shotgun), the NCBI reference of the pC3 sequence (for WGS the contigs covering the *shvR-afc* region are indicated) of the strain used in the alignments, the isolation source, the reference of the sequencing/isolation study (for strains without this reference the BioSample number is given), and the MLST ID that identifies the strain (pubmlst.org/bcc) are indicated.
(DOCX)

S1 Movie Related to Fig 2A. Visualisation of Δ*shvR* bacteria internalised by macrophages. Movie through the sequential stacks (1 μm distance) of a mCherry positive macrophage of a Tg(*mpeg1*:*mCherry-F*) embryo injected with Δ*shvR* expressing eGFP, imaged over the yolk region (the corresponding confocal stack image is presented in Fig 2A).
(AVI)

S2 Movie Related to Fig 4. Real time progression of infection of Δ*shvR* bacteria after rhamnose-induced expression of *shvR*. Movie of a representative Tg(*mpx*:*eGFP*) embryo injected with *ΔshvR*(P*rhaB*:*shvR*) (red) during a 24 h time course at 1 h intervals, starting 3 h after injection of rhamnose in the circulation (27 h after injection of bacteria) using confocal time lapse imaging. Green and red channels were merged using Fluoview (83 images stacked per time point at 1μm distance). The images were rotated, and embryo contour, time indication and scale bar (100 μm) were added in ImageJ. Representative of 5 time lapse videos.
(AVI)
