# Supplementary figures and images for: The afc antifungal activity cluster, which is under tight regulatory control of ShvR, is essential for transition from intracellular persistence of Burkholderia cenocepacia to acute pro-inflammatory infection
Source: PLoS Pathog. 2018 Dec 4;14(12):e1007473. doi: 10.1371/journal.ppat.1007473 (PMC6301696; doi:10.1371/journal.ppat.1007473)

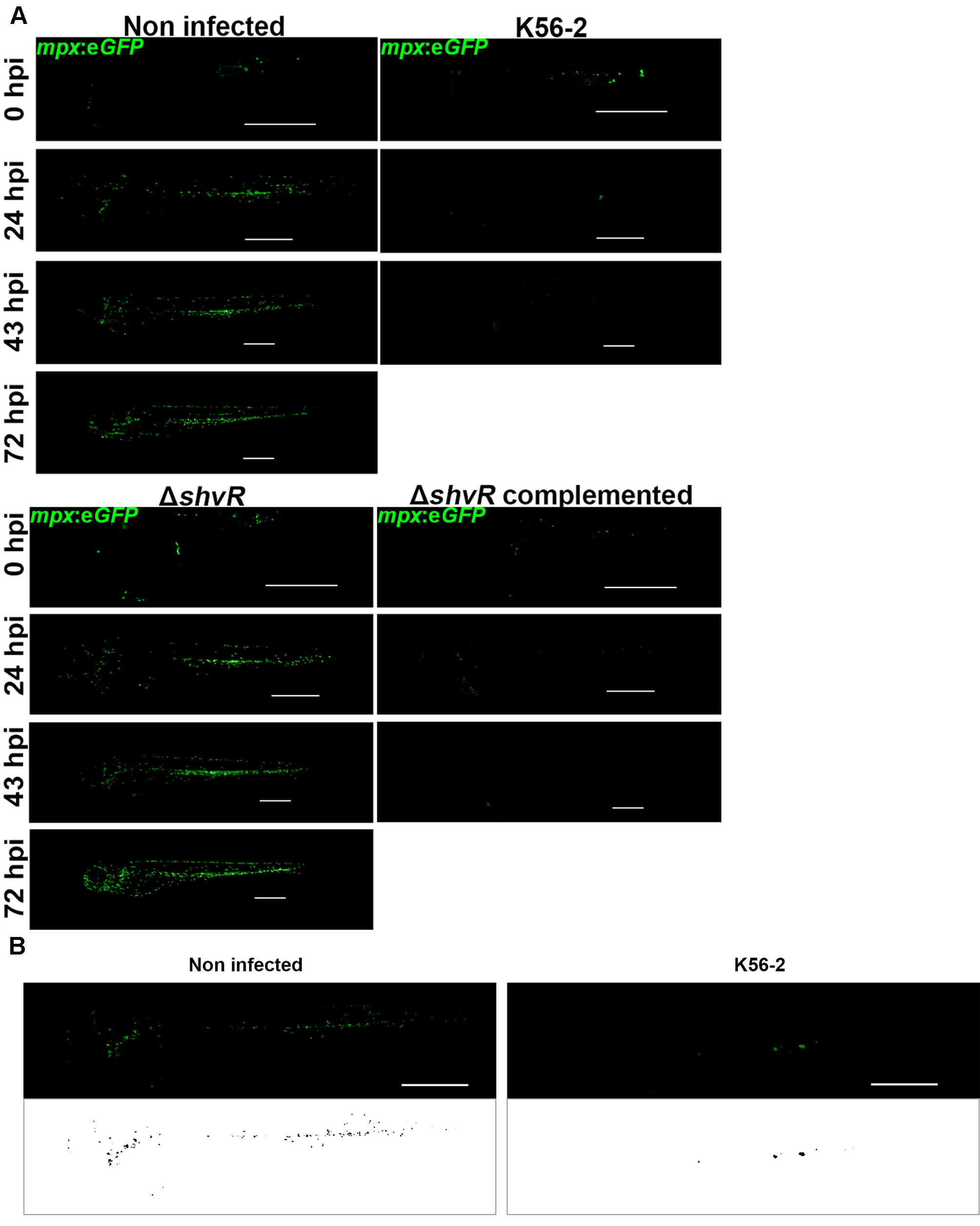

Supplement: S1 Fig — (A) Representative images at 0, 24, 43 and 72 hpi of Tg(mpx:eGFP) embryos, non-infected and infected with B. cenocepacia K56-2, ΔshvR, and the complemented ΔshvR mutant. Scale bars, 50 μm. (B) Representative images of binary conversion of fluorescent microscopy images of Tg(mpx:eGFP) embryos, non-infected and infected with K56-2. Scale bars, 50 μm. (TIF) [file ppat.1007473.s001.tif]

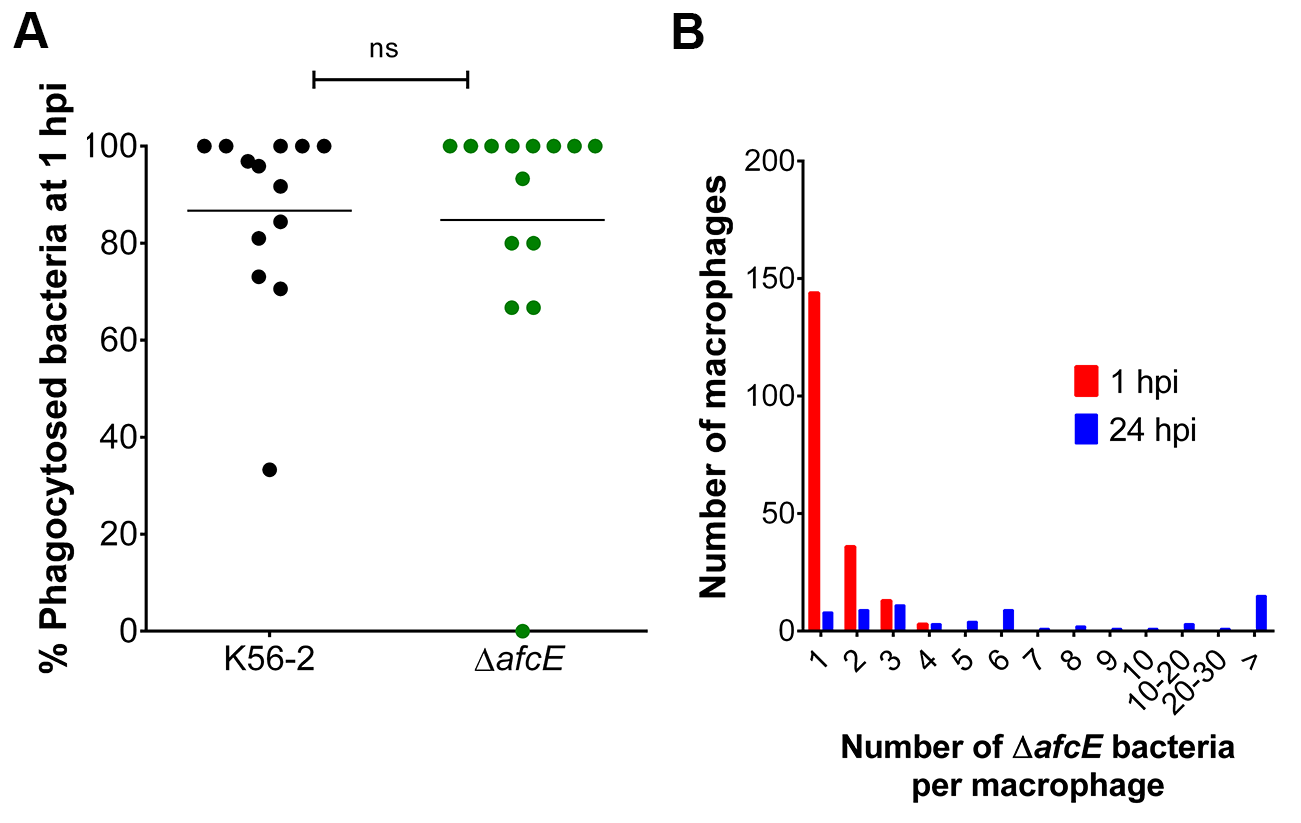

Supplement: S2 Fig — (A) Quantification of intracellular ΔafcE bacteria in PFA-fixed embryos at 1 hpi over the yolk as indicated in Fig 2A for ΔshvR using confocal microscopy. Each data point represents an individual Tg(mpeg1:mCherry-F) transgenic embryo injected intravenously with wildtype B. cenocepacia K56-2 (black dots, n = 13) or ΔafcE (red dots, n = 14) expressing eGFP. Data represent the same experiment as shown in Fig 2B, with the same values for K56-2. Counts per embryo are presented as percentage of internalized bacteria relative to total numbers of bacteria in the yolk sac region. A total of 220 and 122 bacteria were counted for K56-2 and ΔafcE, respectively, in 15 embryos. Unpaired t-test, with mean 86.7 ± 5.3 and 84.7 ± 7.3, respectively, p-value 0.84. Data are representative of two independent experiments. (B) Quantification of the number of intracellular ΔafcE bacteria per macrophage in PFA-fixed embryos at 1 and 24 hpi, presented as frequency distribution histogram. Data represent the same experiment as shown in Fig 2E. The graph represents a total of 197 (14 embryos) and 68 macrophages (12 embryos), at 1 hpi (mean: 1.4 ± 0.07 bacteria per macrophage) and 24 hpi (10.1 ± 1.3), respectively. Mann Whitney test p < 0.0001. Representative of 2 experiments. (TIF) [file ppat.1007473.s002.tif]

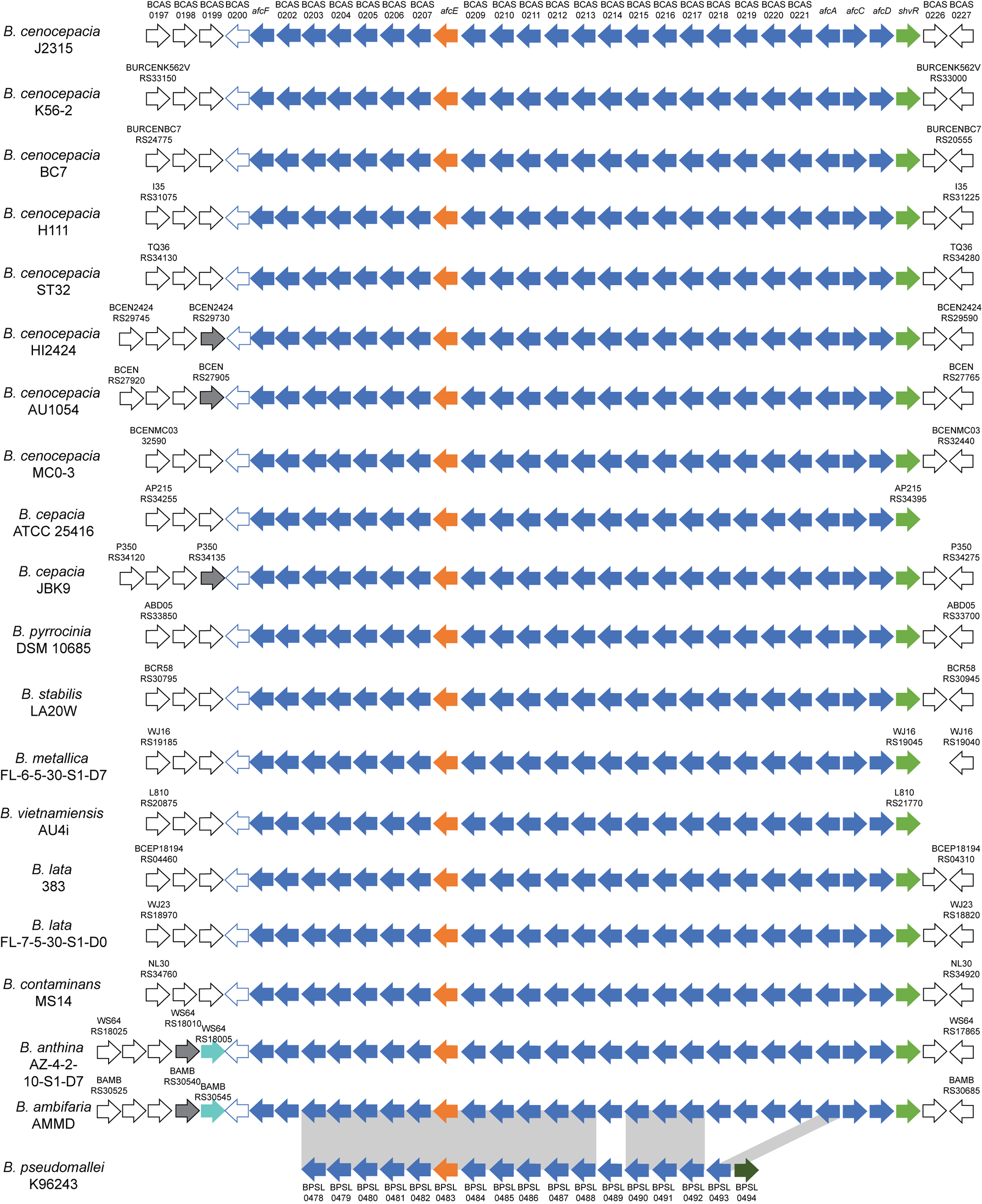

Supplement: S3 Fig — Bioinformatics analysis of the afc cluster and flanks reveals a similar organization in all the 10 species encoding shvR, with variations in the flanking genes. In B. pseudomallei chromosome 1 a similarly structured cluster is present, although it is shorter and regulated by a LTTR that differs from shvR (dark green arrow). Homology between the afc cluster and the genes in B. pseudomallei is indicated with grey shades. shvR is indicated in green, afcE in orange and the other genes of the afc cluster in blue. Blue empty arrow indicates a putative coding sequence that has not been annotated in all strains (BCAS0200). Black arrows indicate the flanking genes of the shvR-afc cluster: the C4-dicarboxylate ABC transporter operon (BCAS197-BCAS199) on the left, and a hydrolase-encoding gene (BCAS0226) and an MFS transporter (BCAS0227) on the right side. Grey arrows represent a 6-phosphogluconolactanase encoding gene and a chemotaxis protein is marked with a light blue arrow. Schematic representation of the genes is not to scale. (TIF) [file ppat.1007473.s003.tif]

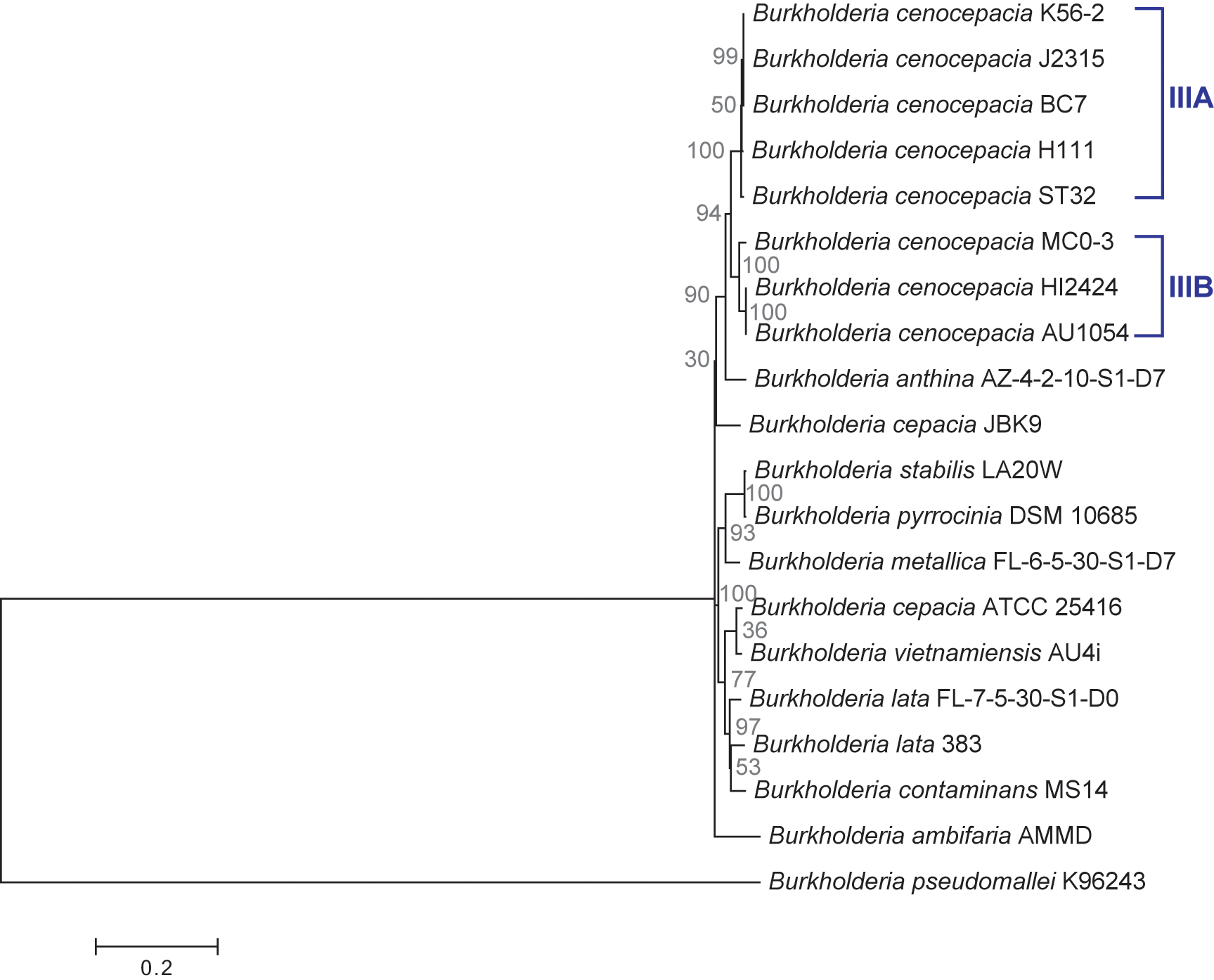

Supplement: S4 Fig — Maximum likelihood phylogeny tree was inferred based on the alignment of afcE nucleotide sequences in Bcc and B. pseudomallei (bootstrap 1000 replicates). The distances for nucleotide data were inferred using the Tamura 3-parameter model, with gamma distribution (5 rate categories) and 40% invariable sites. Scale bar indicates 0.2 substitutions per site. (TIF) [file ppat.1007473.s004.tif]

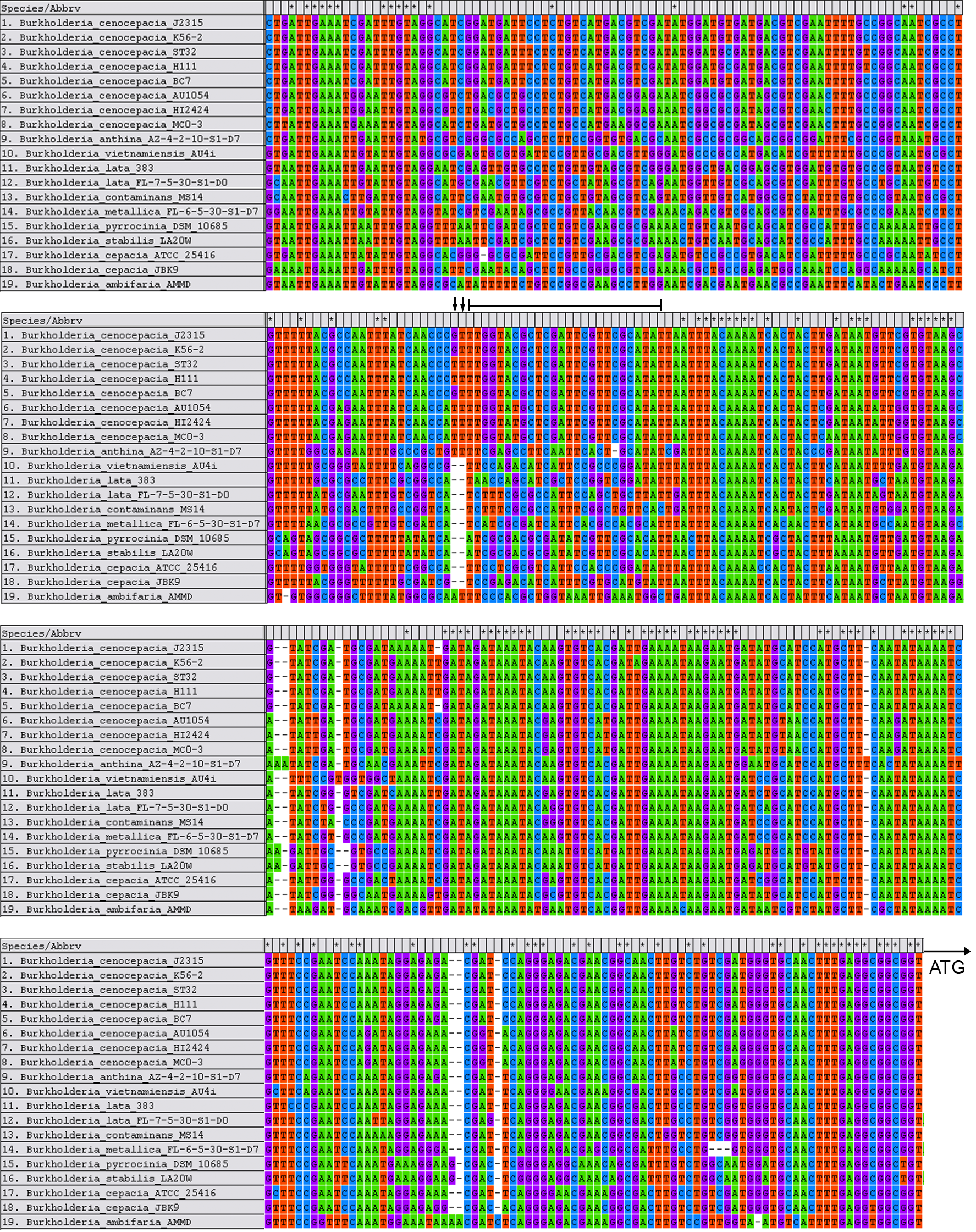

Supplement: S5 Fig — The sequences shown are only 268 bp of the 617 bp intergenic region between afcD and shvR. The nucleotide sequence located upstream of shvR has several SNPs in the other 18 shvR encoding Bcc strains compared to B. cenocepacia J2315 (see Fig 8). Between the different B. cenocepacia strains the similarity ranges from 87 to 99% identity (the weakest similarities are observed for the strains HI2424, MC0-3 and AU1054). Compared with the other Bcc species, the similarities range from 76% to 81%. The 2-nucleotide gap at -238 bp from the translation start site (ATG) is indicated with black vertical arrows. The black bar indicates a short highly non-homologous region compared to the conserved sequence found in the B. cenocepacia J2315. (TIF) [file ppat.1007473.s005.tif]

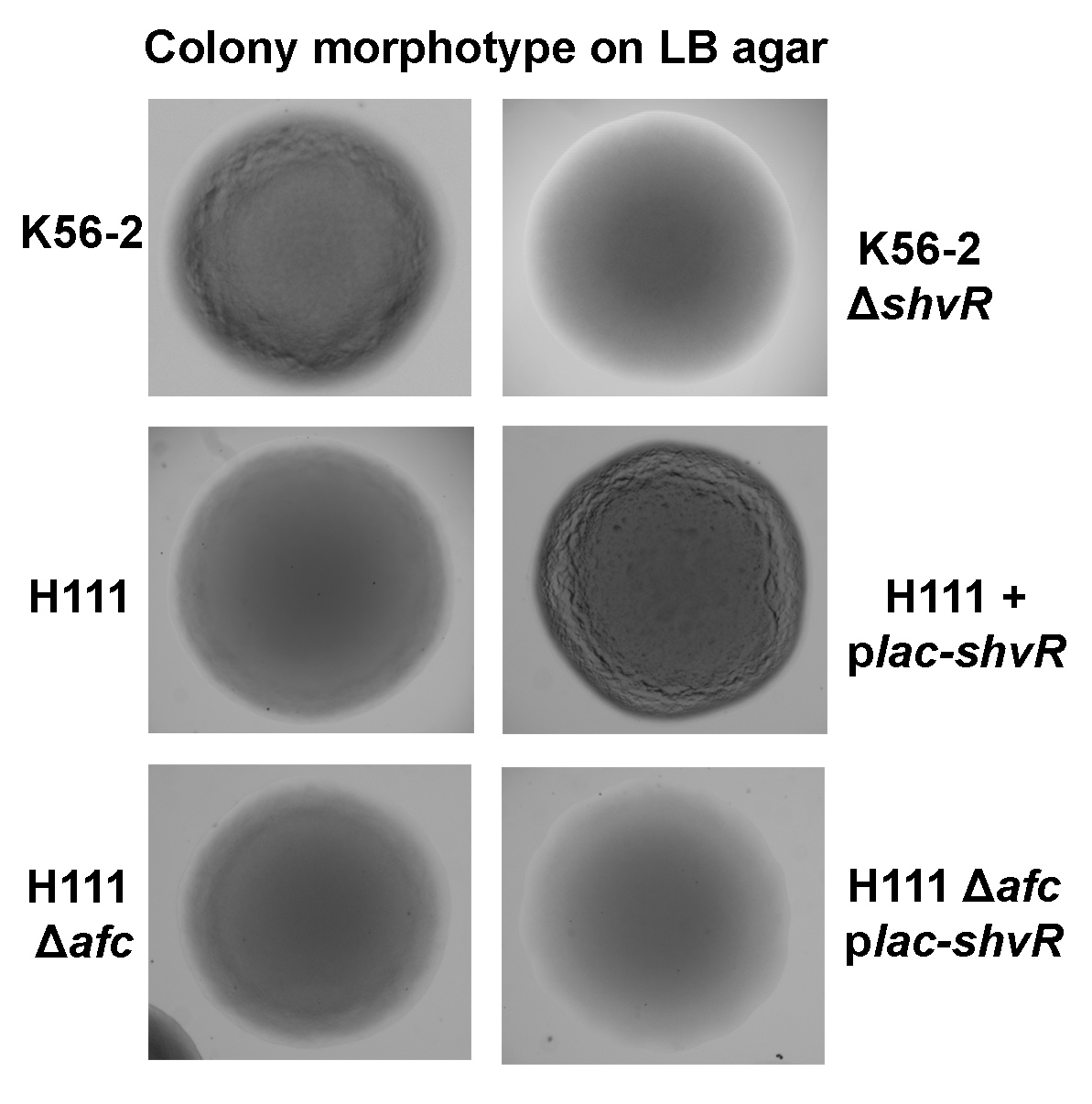

Supplement: S6 Fig — Effect of expression of Plac-shvR on morphology of individual colonies, visualised using an AZ100 microscope. (TIF) [file ppat.1007473.s006.tif]
