# Supplementary material for: The afc antifungal activity cluster, which is under tight regulatory control of ShvR, is essential for transition from intracellular persistence of Burkholderia cenocepacia to acute pro-inflammatory infection
Source: PLoS Pathog. 2018 Dec 4;14(12):e1007473. doi: 10.1371/journal.ppat.1007473 (PMC6301696; doi:10.1371/journal.ppat.1007473)
Supplement: S1 Table — Bacterial strains, plasmids and primers used in this study. (DOCX) [file ppat.1007473.s007.docx]

S1 Table. Related to experimental procedures. Bacterial strains, plasmids and primers used in this study.

| Bacterial strains | Description | Reference |
| --- | --- | --- |
| *Escherichia coli* DH5α | Φ80*lac*ZΔM15 Δ*lac*U169 *endA1* *recA1* *hsdR17* *supE44* *thi*-1 *gyrA96* *relA1* |  |
| *Burkholderia cenocepacia* K56-2 | CF sputum isolate (Canada); ET12 lineage; cblA+recA subgroup | [1] |
| Δ*shvR* | Derivative of K56-2 with a precise deletion of *shvR* | [2] |
| Δ*afcE* | Derivative of K56-2 with a precise, non-polar, deletion of *afcE* | [3] |
| *Burkholderia cenocepacia* H111 | CF sputum isolate | [4] |
| Δ*afc* | Derivative of H111 with a precise deletion of *afc* | This study |
| Plasmids |  |  |
| pBBR1MCS-3 | Broad host range, mobilizable plasmid pBBR1CM with pBluescript II KS-*lacZα*-polylinker; Tet^R^ | [5] |
| pIN29 | DSRed reporter-containing plasmid, pBBR-derived; Cm^R^ | [6] |
| pSAT1:mCherry-MCS-nVenus (pE3370) | mCherry reporter-containing vector, pSAT-derived; Amp^R^ | [7] |
| pIN233 | mCherry reporter-containing plasmid, pIN29-derived; Cm^R^ | This study |
| pCR11 | Cloning vector pMR10-derived; Cm^R^ | M. Kovach |
| pIN298 | mCherry reporter-containing plasmid, pCR11-derived; Cm^R^  Referred to as mCherry_pCR11_ | This study |
| pIN307 | P*shvR*:*shvR* expressing plasmid, pIN29-derived; Cm^R^ | This study |
| pIN308 | P*shvR*:*shvR* expression plasmid, mCherry marker, pIN298-derived; Cm^R^  Referred to as P*shvR*:*shvR*; mCherry_pCR11_ | This study |
| pIN177 | Cloning vector without *mobA* and *oriT*, containing *tac* promoter sequence, pBBR-derived; Cm^R^ | This study |
| pSCrhaB2 | *rhaRS*–P*rhaB*, pBBR1-derived, Tp^R^ | [8] |
| pIN299 | *rhaRS*–P*rhaB*, pBBR-derived; Cm^R^  Referred to as P*rhaB*- | This study |
| pIN309 | *rhaRS*–P*rhaB* expressing *shvR* coding region, pIN299-derived; Cm^R^ | This study |
| pIN310 | *rhaRS*–P*rhaB* expressing *shvR* coding region, mCherry marker, pIN299-derived; Cm^R^  Refered to as P*rhaB*:*shvR* | This study |
| pIN311 | *rhaRS*–P*rhaB* expressing afcE coding region, pIN299-derived; Cm^R^  Referred to as P*rhaB*:*afcE* | This study |
| pIN72 | DSRed reporter-containing vector, pBBR1MCS-3-derived; Tet^R^  Referred to as DSRed_pBBR_ | [6] |
| pBBRS0208 | P*lac*:*afcE* expressing plasmid, pBBR-derived; Tet^R^ | [3] |
| pBBRDSRedS0208 | *afcE* expressing plasmid, P*tac*:DsRed marker, pBBR-derivative; Tet^R^  Referred to as P*lac*:*afcE*;DSRed_pBBR_ | This study |
| pIN301 | eGFP reporter-containing plasmid, pBBR-derived; Cm^R^ | [9] |
| pBBR1MCS | pBBR cloning vector; Cm^R^ | [10] |
| P*lac*-*shvR*_pBBR_ | *shvR*_H111_ expression plasmid, with *shvR* under control of *lac* promoter. pBBR-MCS1, Cm^R^ | This study |
| pSHAFT2-FRT | FRT site containing plasmid, pSHAFT2-derived; Cm^R^ | [11] |
| pEX18Tp-FRT | FRT site containing plasmid, pEX18Tp-derived; Tp^R^ | [11] |
| pSHAFT2-FRT-afcUP | Upstream flanking region of the *afc* cluster, pSHAFT2-FRT-derived; Cm^R^ | This study |
| pEX18Tp-FRT-afcDOWN | Downstream flanking region of the *afc* cluster, pEX18Tp-FRT-derived; Tp^R^ | This study |
| pBBR5::FLP | Flippase cassette containing plasmid, pBBR1MCS-5-derived; Gm^R^ | [12] |
| pBBR5::FLP-TetAR | Tetracycline resistance-containing plasmid, pBBR5::FLP-derived; Tet^R^ | This study |
| Primers |  |  |
| mCherry 3 | 5’-GGAATTCCATATGGTGAGCAAGGGCGAGGAG-3’ | This study |
| mCherry 4 | 5’- GCTCTAGACTACTTGTACAGCTCGTCCATGCC-3’ | This study |
| pshvRXhoI for | 5’-AATTCTCGAGGAATTTTCCGCCCGACATGCGC-3’ | This study |
| shvRXbaI rev | 5’-GTTCTAGACTATCCGACGCGATACATCGGC-3’ | This study |
| shvRNdeI for | 5’-GCTTCATATGGCTAATGTGAGATTGGCAAAGC-3’ | This study |
| 0208_NdeI for | 5’-GCTTCATATGAGCGCATACAAGG-3’ | This study |
| 0208_XbaI rev | 5’-GTTCTAGAATCATTCCACCGCGGC-3’ | This study |
| shvRHindFor | 5’-GCGCAAGCTTCGGCGGATTATGCATTTCCG-3’ | This study |
| shvRBamRev | 5’-GCGCGGATCCCTATCCGACGCGATACATCG-3’ | This study |
| upXhoF | 5’-GCGCCTCGAGTTCTGATCTTCCTCGTGCTC-3’ | This study |
| upBglIIR | 5’-GCGCAGATCTGCAACGCAATCAGAACACC-3’ | This study |
| downpstF | 5’-GCGCCTGCAGATTCATCTTGACGGTCGTCG-3’ | This study |
| downBamR | 5’-GCGCGGATCCTTGATCGTACTGGCTGAAGT-3’ | This study |
| AFCDELcheckFor | 5’-GTACATCAACATCATCCAGG-3’ | This study |
| AFCDELcheckRev | 5’-GGGTTGATAAATTGGCGTAA-3’ | This study |
| tetPst_F | 5’-GCGCCTGCAGTCAGCGATCGGCTCGTTGCC-3’ | This study |
| tetPst_R | 5’-GCGCCTGCAGTCAATCGTCACCCTTTCTCG-3’ | This study |
| qRT-PCR primers |  |  |
| ppial for | 5’-ACACTGAAACACGGAGGCAAAG-3’ |  |
| ppial rev | 5’-CATCCACAACCTTCCCGAACAC-3’ |  |
| cxcl8 for | 5’-TGTGTTATTGTTTTCCTGGCATTTC-3’ |  |
| cxcl8 rev | 5’-GCGACAGCGTGGATCTACAG-3’ |  |
| il1b for | 5’-GAACAGAATGAAGCACATCAAACC-3’ |  |
| il1b rev | 5’-ACGGCACTGAATCCACCAC-3’ |  |

**References**

1. Darling P, Chan M, Cox AD, Sokol PA. Siderophore production by Cystic Fibrosis isolates of *Burkholderia cepacia*. Infect Immun. 1998;66: 874–877.

2. O’Grady EP, Nguyen DT, Weisskopf L, Eberl L, Sokol PA. The *Burkholderia cenocepacia* LysR-Type transcriptional regulator ShvR influences expression of quorum-sensing, protease, type II secretion, and afc genes. J Bacteriol. 2011;193: 163–176. doi:10.1128/JB.00852-10

3. Subramoni S, Nguyen DT, Sokol PA. *Burkholderia cenocepacia* ShvR-Regulated Genes That Influence Colony Morphology, Biofilm Formation, and Virulence. Infect Immun. 2011;79: 2984–2997. doi:10.1128/IAI.00170-11

4. Romling U, Fiedler B, Bosshammer J, Grothues D, Greipel J, von der Hardt H, et al. Epidemiology of chronic *Pseudomonas aeruginosa* infections in cystic fibrosis. Concise Commun lID. 1994;170: 1616–1621.

5. Kovach ME, Elzer PH, Steven Hill D, Robertson GT, Farris MA, Roop RM, et al. Four new derivatives of the broad-host-range cloning vector pBBR1MCS, carrying different antibiotic-resistance cassettes. Gene. 1995;166: 175–176. doi:10.1016/0378-1119(95)00584-1

6. Vergunst AC, Meijer AH, Renshaw SA, O’Callaghan D. *Burkholderia cenocepacia* creates an intramacrophage replication niche in zebrafish embryos, followed by bacterial dissemination and establishment of systemic infection. Infect Immun. 2010;78: 1495–1508. doi:10.1128/IAI.00743-09

7. Lee LY, Wu FH, Hsu CT, Shen SC, Yeh HY, Liao DC, et al. Screening a cDNA Library for Protein-Protein Interactions Directly in Planta. Plant Cell. 2012;24: 1746–1759. doi:10.1105/tpc.112.097998

8. Cardona ST, Valvano MA. An expression vector containing a rhamnose-inducible promoter provides tightly regulated gene expression in *Burkholderia cenocepacia.* Plasmid. 2005;54: 219–228. doi:10.1016/j.plasmid.2005.03.004

9. Mesureur J, Feliciano JR, Wagner N, Gomes MC, Zhang L, Blanco-Gonzalez M, et al. Macrophages, but not neutrophils, are critical for proliferation of *Burkholderia cenocepacia* and ensuing host-damaging inflammation. Tobin DM, editor. PLOS Pathog.; 2017;13: e1006437. doi:10.1371/journal.ppat.1006437

10. Kovach ME, Phillips RW, Elzer PH, Roop RM, Peterson KM. pBBR1MCS: a broad-host-range cloning vector. Biotechniques. 1994;16: 800–2. http://www.ncbi.nlm.nih.gov/pubmed/8068328

11. Agnoli K, Freitag R, Gomes MC, Jenul C, Suppiger A, Mannweiler O, et al. The Use of Synthetic Hybrid Strains to Determine the Role of Replicon 3 in Virulence of the *Burkholderia cepacia* Complex. Appl Environ Microbiol. 2017;83: AEM.00461-17. doi:10.1128/AEM.00461-17

12. Agnoli K, Schwager S, Uehlinger S, Vergunst A, Viteri DF, Nguyen DT, et al. Exposing the third chromosome of *Burkholderia cepacia* complex strains as a virulence plasmid. Mol Microbiol. 2012;83: 362–378. doi:10.1111/j.1365-2958.2011.07937.x
