# Supplementary material for: The afc antifungal activity cluster, which is under tight regulatory control of ShvR, is essential for transition from intracellular persistence of Burkholderia cenocepacia to acute pro-inflammatory infection
Source: PLoS Pathog. 2018 Dec 4;14(12):e1007473. doi: 10.1371/journal.ppat.1007473 (PMC6301696; doi:10.1371/journal.ppat.1007473)
Supplement: S2 Table — For each strain the genome status (WGS stands for Whole Genome Shotgun), the NCBI reference of the pC3 sequence (for WGS the contigs covering the shvR-afc region are indicated) of the strain used in the alignments, the isolation source, the reference of the sequencing/isolation study (for strains without this reference the BioSample number is given), and the MLST ID that identifies the strain (pubmlst.org/bcc) are indicated. (DOCX) [file ppat.1007473.s008.docx]

S2 Table. Related to Figure 8. *Burkholderia cepacia* complex strains and outgroup species used in this study.

| *Burkholderia cepacia* complex | Genome status | pC3 NCBI Reference Sequence | Isolation source | Reference/BioSample | MLST ID |
| --- | --- | --- | --- | --- | --- |
| *Burkholderia ambifaria* AMMD (LMG 19182) | Complete | NC_008392 | Pea rhizosphere | [1] | 81 |
| *Burkholderia ambifaria* MC40-6 (LMG 23451) | Complete | NC_010557 | Maize rhizosphere | [2] | 563 |
| *Burkholderia anthina* strain AZ-4-2-10-S1-D7 | WGS | NZ_CM003769 | Soil | SAMN03449213 |  |
| *Burkholderia arboris*  (LMG 14939) | Not sequenced |  | Cystic fibrosis patient | [3] | 114 |
| *Burkholderia cenocepacia* AU1054 (LMG 24506) | Complete | NC_008062 | Sputum sample of a patient with cystic fibrosis | [3] | 198 |
| *Burkholderia cenocepacia* BC7 (LMG 18826) | WGS | ALIZ02000045  ALIZ02000152  ALIZ02000195  ALIZ02000230 | Sputum sample of a patient with cystic fibrosis | [3,4] | 35 |
| *Burkholderia cenocepacia* H111 (LMG 23991) | Complete | NZ_HG938372 | Sputum sample of a patient with cystic fibrosis | [5] | 618 |
| *Burkholderia cenocepacia* HI2424 (LMG 24507) | Complete | NC_008544 | Onion field, non-rhizospheric | [3,6] | 197 |
| *Burkholderia cenocepacia* J2315 (LMG 16656) | Complete | NC_011002.1 | Cystic fibrosis patient | [7,8] | 28 |
| *Burkholderia cenocepacia* K56-2 (LMG 18863) | WGS | NZ_ALJA02000017 | Sputum sample of a patient with cystic fibrosis | [3] | 34 |
| *Burkholderia cenocepacia* MC0-3 (LMG 24308) | Complete | NC_010512 | Maize rhizosphere | SAMN02598404 | 688 |
| *Burkholderia cenocepacia* strain ST32 | Complete | NZ_CP011919 | Sputum from a cystic fibrosis patient | SAMN03323790 |  |
| *Burkholderia cepacia* ATCC 25416 (LMG 1222) | Complete | NZ_CP012983 | Onion | [9] | 10 |
| *Burkholderia cepacia* GG4 | Complete | -- | Ginger rhizosphere | [10,11] |  |
| *Burkholderia cepacia* JBK9 | Complete | NZ_CP013732 | Garlic farming soil | SAMN02680268 |  |
| *Burkholderia contaminans* strain MS14 | Complete | NZ_CP009745 | Disease suppressive soil | [12] |  |
| *Burkholderia diffusa* strain RF2-non-BP9 | Complete | NZ_CP013364 | Soil | SAMN03449129 |  |
| *Burkholderia dolosa* AU0158 (LMG 24508) | Complete | NZ_CP009794 | Cystic fibrosis patient | SAMN03144971 | 810 |
| *Burkholderia dolosa* PC543 (LMG 19468) | Complete | NZ_CM002279 | Cystic fibrosis patient | [3] | 2438 |
| *Burkholderia lata* strain 383 (LMG 22485T) | Complete | NC_007509 | Forest soil | [3,13] | 105 |
| *Burkholderia lata* strain FL-7-5-30-S1-D0 | Complete | NZ_CP013405 | Environmental | SAMN03449255 |  |
| *Burkholderia latens* strain AU17928 | Complete | NZ_CP013437 | CF Maxillary Sinus | SAMN03449404 |  |
| *Burkholderia metallica* strain FL-6-5-30-S1-D7 | Complete | NZ_CP013402 | Soil | SAMN03449247 |  |
| *Burkholderia multivorans* ATCC 17616 (LMG 17588) | Complete | NC_010801 | Soil | [3] | 21 |
| *Burkholderia multivorans* ATCC BAA-247 (LMG 13010) | Complete | NZ_CP009830 | Cystic fibrosis patient | [14] |  |
| *Burkholderia multivorans* strain AU1185 | Complete | NZ_CP013432 | Thigh Biopsy | SAMN03449401 |  |
| *Burkholderia multivorans* strain DDS 15A-1 | Complete | NZ_CP008728 | Aerosol sample | SAMN02848467 | 1331 |
| *Burkholderia multivorans* strain MSMB1640WGS | Complete | NZ_CP013468 | Aerosol sample | SAMN03449689 |  |
| *Burkholderia paludis* MSh1 | WGS | NZ_JPGL01000010 | Malaysian Tropical Peat Swamp Forest Soil | [15] | 1940 |
| *Burkholderia pseudomultivorans* strain SUB-INT23-BP2 | Complete | -- | Soil | SAMN03449185 |  |
| *Burkholderia pyrrocinia* strain DSM 10685 (LMG 14191) | Complete | NZ_CP011505 | Soil | [16] |  |
| *Burkholderia seminalis* strain FL-5-4-10-S1-D7 | Complete | NZ_CP013399 | Soil | SAMN03449243 |  |
| *Burkholderia stabilis* strain ATCC BAA-67 (LMG 14294) | Complete | NZ_CP016444 | Sputum from a cystic fibrosis patient | [3,17] | 54 |
| *Burkholderia stabilis* strain LA20W | WGS | NZ_DF978419 | Cellulosic biomass | [18] |  |
| *Burkholderia stagnalis* strain MSMB735WGS | Complete | NZ_CP013460 | Soil | SAMN03449637 |  |
| *Burkholderia territorii* strain MSMB2203WGS | Complete | NZ_CM004193 | Soil | SAMN03449584 |  |
| *Burkholderia territorii* strain RF8-non-BP5 | Complete | NZ_CP013367 | Soil | SAMN03449137 |  |
| *Burkholderia ubonensis* Bu | WGS | -- | Unknown | SAMN02470672 |  |
| *Burkholderia ubonensis* MSMB22 | Complete | -- | Soil | [19] | 2099 |
| *Burkholderia* *vietnamiensis* AU4i | WGS | NZ_ASSI01000089  NZ_ASSI01000095  NZ_ASSI01000114  NZ_ASSI01000299 | Pea rhizosphere | SAMN02469984 |  |
| *Burkholderia* *vietnamiensis* G4 (LMG 22486) | Complete | NC_009254 | Industrial | [3] | 64 |
| *Burkholderia vietnamiensis* LMG 10929 | Complete | NZ_CP009632 | Rice rhizosphere | [3,19] | 69 |
| *Burkholderia vietnamiensis* strain AU1233 | Complete | NZ_CP013434 | Blood sample from a cystic fibrosis patient | SAMN03449402 |  |
| Outgroups |  |  |  |  |  |
| *Burkholderia pseudomallei* K96243 | Complete | NC_006350 (chr1) | Human case of melioidosis | [20] | 172 |
| *Burkholderia thailandensis* E264 (LMG 20219) | Complete | -- | Rice field sample | [21] |  |
| *Ralstonia pickettii* 12J | Complete | -- | Unknown | SAMN02598396 |  |

**References**

1. Coenye T, Mahenthiralingam E, Henry D, LiPuma JJ, Laevens S, Gillis M, et al. *Burkholderia ambifaria* sp. nov., a novel member of the *Burkholderia cepacia* complex including biocontrol and cystic fibrosis-related isolates. Int J Syst Evol Microbiol. 2001;51: 1481–1490. doi:10.1099/00207713-51-4-1481

2. Rose H, Baldwin A, Dowson CG, Mahenthiralingam E. Biocide susceptibility of the *Burkholderia cepacia* complex. J Antimicrob Chemother. 2009;63: 502–510. doi:10.1093/jac/dkn540

3. Baldwin A, Mahenthiralingam E, Thickett KM, Honeybourne D, Maiden MCJ, Govan JR, et al. Multilocus Sequence Typing Scheme That Provides Both Species and Strain Differentiation for the *Burkholderia cepacia* Complex. J Clin Microbiol. 2005;43: 4665–4673. doi:10.1128/JCM.43.9.4665-4673.2005

4. Varga JJ, Losada L, Zelazny AM, Kim M, McCorrison J, Brinkac L, et al. Draft Genome Sequences of *Burkholderia cenocepacia* ET12 Lineage Strains K56-2 and BC7. Genome Announc. American Society for Microbiology (ASM); 2013;1. doi:10.1128/genomeA.00841-13

5. Romling U, Fiedler B, Bosshammer J, Grothues D, Greipel J, von der Hardt H, et al. Epidemiology of chronic *Pseudomonas aeruginosa* infections in cystic fibrosis. Concise Commun lID. 1994;170: 1616–1621.

6. LiPuma JJ, Spilker T, Coenye T, Gonzalez CF. An epidemic Burkholderia cepacia complex strain identified in soil. Lancet. 2002;359: 2002–2003. doi:10.1016/S0140-6736(02)08836-0

7. Govan JRW, Doherty CJ, Nelson JW, Brown PH, Greening AP, Maddison J, et al. Evidence for transmission of *Pseudomonas cepacia* by social contact in cystic fibrosis. Lancet. 1993;342: 15–19. doi:10.1016/0140-6736(93)91881-L

8. Holden MTG, Seth-Smith HMB, Crossman LC, Sebaihia M, Bentley SD, Cerdeno-Tarraga AM, et al. The Genome of *Burkholderia cenocepacia* J2315, an Epidemic Pathogen of Cystic Fibrosis Patients. J Bacteriol. 2009;191: 261–277. doi:10.1128/JB.01230-08

9. Ballard RW, Pallroni NJ, Doudoroff M, Stanier RY. Taxonomy of the Pseudomonads: *Pseudomonas cepacia*, *P. marginata, P. alliicola,* and *P. Caryophylli*. J Gen Microbiol. 1970;60: 199–214.

10. Chan K-G, Atkinson S, Mathee K, Sam C-K, Chhabra SR, Cámara M, et al. Characterization of N-acylhomoserine lactone-degrading bacteria associated with the Zingiber officinale (ginger) rhizosphere: co-existence of quorum quenching and quorum sensing in *Acinetobacter* and *Burkholderia*. BMC Microbiol. BioMed Central; 2011;11: 51. doi:10.1186/1471-2180-11-51

11. Hong KW, Koh CL, Sam CK, Yin WF, Chan KG. Complete genome sequence of *Burkholderia* sp. Strain GG4, a betaproteobacterium that reduces 3-oxo-N-Acylhomoserine lactones and produces different N-acylhomoserine lactones. J. Bact. 2012. p. 6317. doi:10.1128/JB.01578-12

12. Cook RJ, Baker KF. The nature and practice of biological control of plant pathogens. American Phytopathological Society; 1983.

13. Vanlaere E, Baldwin A, Gevers D, Henry D, De Brandt E, LiPuma JJ, et al. Taxon K, a complex within the *Burkholderia cepacia* complex, comprises at least two novel species, *Burkholderia contaminans* sp. nov. and *Burkholderia lata* sp. nov. Int J Syst Evol Microbiol. 2009;59: 102–111. doi:10.1099/ijs.0.001123-0

14. Vandamme P, Holmes B, Vancanneyt M, Coenye T, Hoste B, Coopman R, et al. Occurrence of multiple genomovars of Burkholderia cepacia in cystic fibrosis patients and proposal of *Burkholderia multivorans* sp. nov. Int J Syst Bacteriol. 1997;47: 1188–1200. doi:10.1099/00207713-47-4-1188

15. Ong KS, Aw YK, Gan HM, Yule CM, Lee SM. Draft Genome Sequences of Two Antimicrobial-Producing *Burkholderia* sp. Strains, MSh1 and MSh2, Isolated from Malaysian Tropical Peat Swamp Forest Soil. Genome Announc. 2014;2: e01032-14-e01032-14. doi:10.1128/genomeA.01032-14

16. Kwak Y, Shin JH. Complete genome sequence of *Burkholderia pyrrocinia* 2327T, the first industrial bacterium which produced antifungal antibiotic pyrrolnitrin. J Biotechnol. 2015;211: 3–4. doi:10.1016/j.jbiotec.2015.06.420

17. Revets H, Vandamme P, Van Zeebroeck A, De Boeck K, Struelens MJ, Verhaegen J, et al. *Burkholderia (Pseudomonas) Cepacia* and Cystic Fibrosis: the Epidemiology in Belgium. Acta Clin Belg. Taylor & Francis; 1996;51: 222–230. doi:10.1080/22953337.1996.11718514

18. Habe H, Sato S, Morita T, Fukuoka T, Kirimura K, Kitamoto D. Bacterial production of short-chain organic acids and trehalose from levulinic acid: A potential cellulose-derived building block as a feedstock for microbial production. Bioresour Technol. 2015;177: 381–386. doi:10.1016/j.biortech.2014.11.048

19. Johnson SL, Bishop-Lilly KA, Ladner JT, Daligault HE, Davenport KW, Jaissle J, et al. Complete Genome Sequences for 59 *Burkholderia* Isolates, Both Pathogenic and Near Neighbor. Genome Announc. 2015;3: e00159-15. doi:10.1128/genomeA.00159-15

20. Holden MTG, Titball RW, Peacock SJ, Cerdeno-Tarraga AM, Atkins T, Crossman LC, et al. Genomic plasticity of the causative agent of melioidosis, *Burkholderia pseudomallei.* Proc Natl Acad Sci.; 2004;101: 14240–14245. doi:10.1073/pnas.0403302101

21. Kim HS, Schell MA, Yu Y, Ulrich RL, Sarria SH, Nierman WC, et al. Bacterial genome adaptation to niches: divergence of the potential virulence genes in three *Burkholderia* species of different survival strategies. BMC Genomics. BioMed Central; 2005;6: 174. doi:10.1186/1471-2164-6-174
